# A Checkpoint Reversal Receptor Mediates Bipartite Activation and Enhances CAR T-cell Function

**DOI:** 10.1158/2767-9764.CRC-24-0125

**Published:** 2025-03-31

**Authors:** Daniel Landi, Shoba A. Navai, Rebecca M. Brock, Kristen Fousek, Zeid Nawas, Khaled Sanber, Cynthia Chauvin-Fleurence, Raksha R. Bhat, Shuo Xu, Purna Krishnamurthy, Michelle Choe, Matthew E. Campbell, Jessica S. Morris, Ahmed Z. Gad, Ankita Shree, Alesandra S. Echeandia Marrero, Amr M. Saadeldin, Pretty R. Matthew, Dolores Mullikin, Kevin Bielamowicz, Lyazat Kurenbekova, Angela M. Major, Vita S. Salsman, Tiara T. Byrd, John M. Hicks, Yi Jonathan Zhang, Jason Yustein, Alexandre F. Carisey, Sujith K. Joseph, Nabil Ahmed, Meenakshi Hegde

**Affiliations:** 1 Center for Cell and Gene Therapy, Baylor College of Medicine, Houston, Texas.; 2 Texas Children’s Cancer Center, Baylor College of Medicine, Houston, Texas.; 3 Department of Pediatrics, Baylor College of Medicine, Houston, Texas.; 4 Dan L. Duncan Comprehensive Cancer Center, Baylor College of Medicine, Houston, Texas.; 5 Interdepartmental Program in Translational Biology and Molecular Medicine, Baylor College of Medicine, Houston, Texas.; 6 Development, Disease Models and Therapeutics Graduate Program, Baylor College of Medicine, Houston, TX.; 7 Department of Pathology and Immunology, Baylor College of Medicine, Houston, Texas.; 8 Department of Neurosurgery, Houston Methodist Hospital, Houston, Texas.; 9 Cell & Molecular Biology Department, St. Jude Children’s Research Hospital, Memphis, Tennessee.

## Abstract

The efficacy of chimeric antigen receptor T cells (CART) in solid tumors is limited by immune inhibition. In our study, we observed that effector cytokines mediated the upregulation of the PD-L1 immune checkpoint in primary glioblastoma. To offset the PD-L1 inhibitory signal, we engineered PD-1 checkpoint reversal receptors (CPR) with a CD28 or 41BB costimulatory endodomain and coexpressed them with a first-generation or a CD28-containing second-generation HER2-specific CAR (CPR/CART) using bicistronic vectors. We found that bipartite T-cell activation, by CAR-generated signal 1 and CPR costimulation (signal 2), fine-tuned proinflammatory cytokine release and sustained antitumor activity. Whereas both CPR28 and CPR41BB effectively counteracted the PD-1 signal *in vitro*, CPR41BB, when coexpressed with a first-generation CAR (CARζ/CPR41BB), promoted central memory differentiation following repeat antigenic stimulation. CARζ/CPR41BB T cells formed a robust immune synapse with tumor targets, similar to a 41BB-containing second-generation CART, maintained the favorable metabolic parameters associated with 41BB costimulation, and demonstrated superior antitumor function after adoptive transfer in xenograft models of gioblastoma and metastatic osteosarcoma. Thus, a CPR molecule with 41BB costimulation that curtails PD-1 inhibition and complements CAR signaling to optimize T-cell activation could enhance CART efficacy against solid tumors.

**Significance:**

Enhancing CART function and persistence while balancing immune effector–mediated inflammation is crucial. Using our clinically relevant HER2-CAR platform, we demonstrate that tumor-intrinsic signals like the PD-1/PD-L1 immune checkpoint can be leveraged in CART design to modulate immune synapse and metabolic parameters, improving antitumor function without increasing cytokine production.

## Introduction

Immune checkpoints inhibit T-cell activation to limit autoimmunity, but these pathways are often exploited by solid tumors and regulatory myeloid cells to promote tumor growth and suppress antitumor immunity. Progressive loss of T-cell effector functions in the tumor microenvironment (TME) is partly driven by the upregulation of the PD-1 axis. This phenomenon also extends to adoptively transferred chimeric antigen receptor T cells (CART; refs. 1–3). In preclinical studies, human CART cells have shown susceptibility to PD-1–mediated functional exhaustion (4). PD-1 blockade augments cytokine secretion and survival of CART challenged by serial antigen stimulation (4, 5) and improves tumor control *in vivo* (6). PD-1 primarily impairs T-cell function by dephosphorylating CD28 and other components associated with the T-cell receptor (TCR; ref. 7). Although the precise mechanism of PD-1–mediated CART inhibition is undefined, coexpression of PD-1–dominant negative receptors or chimeric switch receptors with a CD28 endodomain are shown to promote CART activity in preclinical models (4, 8, 9), providing preliminary evidence that CART can be engineered to disrupt the PD-1 axis, which may enhance their function after adoptive transfer (10). More recently, a PD-1–41BB receptor has been studied in TCR-modified T cells (11, 12). However, systematic comparative studies of how chimeric PD-1 receptors with different transmembranes and endodomains affect various CART characteristics are lacking.

The design and components of chimeric receptors are crucial for determining the functional and molecular attributes of CART (13–18). Typically, CD28-containing CART have a differentiated immunophenotype, are highly proliferative, and exhibit potent cytotoxicity (16). In endogenous T cells, the costimulation (signal 2) required for optimal activation is mediated through CD28. Although CD28 costimulation correlates with greater susceptibility to exhaustion in CART, functional exhaustion, particularly after repetitive antigenic stimulation, is observed across CART, independent of the type of costimulatory domain. Preclinical studies comparing activation-induced cell death between CD28- and 41BB-expressing CART cells were either inconclusive or were confounded by other factors (13–17). Analysis of protein phosphorylation events in structurally identical second-generation CARs with either CD28 or 41BB showed that signaling cascades and effector functions cannot be predicted by the costimulatory domain alone; factors like signal strength play a key role (19). CARs have been engineered with costimulatory switch receptors to enhance safety (20) and with dual CD28 and 41BB costimulation to achieve synergy (13, 21). The potential impact of these intricate differences on immune effector cell–mediated toxicity and antitumor activity underscores the need to fine-tune the design and function of chimeric receptors before their clinical use.

In preclinical tumor models, studies have shown that CD28-containing CART cells may have an advantage in the setting of low antigen density or low effector to target ratio (22). In our phase I studies, an FRP5-scFv–based, CD28-containing second-generation HER2-specific CART (CAR28ζ) demonstrated clinical responses in patients with HER2^+^ tumors, but most were not sustained (3, 23–25). Therefore, we focused on modifying the PD-1/PD-L1 (and PD-L2) interaction to enhance CAR28ζ antitumor activity in HER2^+^ tumor models (22, 26, 27). To restrict the PD-1 axis modulation to the tumor site and to boost T-cell function, we developed bicistronic vectors encoding a PD-1 checkpoint reversal receptor (CPR) and a HER2-CAR, with different permutations of signaling domains (CPR/CART). To develop a clinically viable CPR/CART, we studied the impact of CPR on the phenotype of CART derived from patients with glioblastoma (GBM), a profoundly immune-suppressive tumor (28–31), and assessed their effector function against autologous tumor cells with variable HER2 and PD-L1 expressions. We performed a rigorous comparison of the *in vivo* antitumor activity of candidate CPR/CART in two orthotopic models of GBM and found that CPR with 41BB costimulation augmented the efficacy of first-generation HER2-CART (CARζ/CPR41BB) over the second-generation counterparts. We further examined the *in vivo* function of systemically administered CARζ/CPR41BB cells in an orthotopic model of aggressive, metastatic HER2^+^ osteosarcoma (32). In this study, we describe a systematic evaluation of CPR/CART designs and the collective effect of bipartite signaling on overall CART quality and function. In our studies, the T-cell activation dynamics and metabolic parameters imparted by CPR41BB-enhanced antitumor function, supporting clinical testing of CARζ/CPR41BB in patients with HER2^+^ tumors.

## Materials and Methods

### Study design

The goal of this study was to develop a translatable approach for safely enhancing the antitumor activity of CART targeting a tumor-associated antigen through modulation of the PD-1 axis. The study concurrently aimed to understand the mechanistic and functional variances arising from differences in CAR and CPR designs to better predict and optimize T-cell behavior after adoptive transfer. Building on our prior experience with early clinical testing, we evaluated the impact of CPR on various effector functions of HER2-specific CART using preclinical models of GBM. We then extended our studies to an osteosarcoma model to examine questions pertinent to systemic administration of CART. We generated candidate CPR/CART using primary T cells obtained from healthy donors and from patients with GBM for phenotypic and functional characterization. In validation assays requiring extended experimental conditions, healthy donor T cells were used because of limited availability of patient samples. CPR/CART cells were normalized for transduction where indicated. All *in vitro* experiments were conducted in sample duplicates or triplicates using multiple biological replicates. For *in vivo* studies, mice were randomized as applicable, and sample size was powered to compare the changes from baseline with a fixed follow-up time point(s) among different groups to control treatment with CAR28ζ cells. Survival data were analyzed using the Kaplan–Meier method. Investigators were not blinded. All research involving human samples and animal models were conducted in accordance with institutional guidelines.

### Blood donors, GBM samples, and tumor cell lines

Peripheral blood samples were obtained from healthy donors according to a Baylor College of Medicine (BCM) Institutional Review Board–approved research protocol. Tumor tissue and peripheral blood samples were collected from patients with GBM using research protocols approved by the BCM and Houston Methodist Hospital institutional review boards. Written informed consent was obtained from all donors, and research studies were conducted in accordance with the Declaration of Helsinki. All human cell lines (U373, RRID: CVCL_2219; LN229, RRID: CVCL_0393; Raji, RRID: CVCL_0511, 143B, RRID: CVCL_2270; and THP1, RRID: CVCL_0006) were purchased from the ATCC and authenticated using short tandem repeat analysis annually or as indicated. All cell lines were regularly tested for *Mycoplasma* (once a month or as indicated) using Cambrex MycoAlert Mycoplasma Detection Assay. Primary GBM cells and GBM and osteosarcoma cell lines were maintained in DMEM (Cytiva Life Sciences) with 10% FCS (Cytiva Life Sciences) and 1× GlutaMAX (Thermo Fisher Scientific). Raji and THP1 cells were maintained in RPMI (Thermo Fisher Scientific) with 10% FCS (Cytiva Life Sciences) and 1× GlutaMAX (Thermo Fisher Scientific). All tumor cell lines used for experiments were passaged <10 times, and primary GBM cells were passaged <5 times. Tumor targets for functional evaluation of CPR/CART were chosen based on their *in vitro* and *in vivo* growth kinetics, experimental question, HER2 and PD-L1/PD-L2 expression, and the assay used.

### Generation of PD-L1 knockout LN229 cells

PD-L1 knockout (KO) LN229 cells were generated using the CRISPR-mediated PD-L1 (CD274) Human Gene Knockout Kit (KN213071, OriGene) according to the manufacturer’s instructions. Briefly, WT LN229 cells were transfected with the guide RNA vector and puromycin resistance donor plasmid using GeneJuice (Sigma-Aldrich). We cultured transfected cells in puromycin for 2 to 3 weeks to select for resistant cells and then incubated the cells overnight with IFN-γ (40 ng/mL; BioLegend) to induce PD-L1 expression. PD-L1–null LN229 cells were isolated using a fluorescence-activated cell sorter (FACSAriaIII, BD Biosciences). Loss of PD-L1 expression was confirmed by reincubating with IFN-γ for 24 hours and subsequently assessing with flow cytometry as described below.

### Design of CAR/CPR transgenes

The FRP5-scFv–derived CD28-containing HER2-CAR was previously described (33, 34). Truncated PD-1 (PD-1_TR_) and all CPR molecules consisted of the native PD-1 extacellular domain and the natural leader sequence. The sequence for human PD-1 was obtained from the Research Collaboratory for Structural Bioinformatics Protein Data Bank. In PD-1_TR_, the endodomain was truncated at the Immunoreceptor tyrosine-based inihibitory motif and Immunoreceptor tyrosine-based activation motif motifs to neutralize immunosuppressive effects. CPR molecules with CD28 and 41BB cytoplasmic domains contained CD28 and CD8α transmembrane domains, respectively. The CAR and CPR extracellular, transmembrane, and cytoplasmic domains were assembled on Clone Manager (Sci-Ed, RRID:SCR_014521). A CPR41BB molecule also included the sequence for mEmerald-GFP expression. For coexpression, CAR and CPR were separated by a retroviral 2A sequence (35). The transgene was codon optimized and synthesized by GeneArt (Thermo Fisher Scientific). The bicistronic transgene for each CAR/CPR cassette was subcloned into an SFG retroviral vector (36) and verified by pyrosequencing (Epoch).

### Retroviral transduction of cells and culture conditions

The retroviral supernatant was produced by transfection of 293T cells, and anti-CD3 (Thermo Fisher Scientific, 14-0037-82, RRID: AB_467057) and anti-CD28 (BD Biosciences, 348040, RRID: AB_400367) activated T cells were transduced with CAR/CPR as described (34, 37). Gene-modified T cells were expanded and maintained in culture media (RPMI, Thermo Fisher Scientific) supplemented with IL-7 and IL-15 (Pepro Tech). Tumor cells were transduced with HER2, PD-L1, or eGFP-Firefly luciferase (Ffluc)-encoding transgenes as described (38).

### Flow cytometry

Cells were analyzed on Accuri C6 (BD Biosciences, RRID: SCR_019591), Gallios (Beckham-Coulter, RRID: SCR_016702), and FACSCanto-II (BD Biosciences, RRID: SCR_018056) flow cytometers. Flow cytometry beads or single-stain control samples were used for calibration and compensation for multicolor flow panels. Unstained tumor cells were used to establish gating, and isotype antibodies were used when appropriate to exclude nonspecific binding. Adequate cell viability was confirmed prior to each flow cytometry experiment. For detection of PD-L1 expression in tumor cells, 25,000 to 100,000 cells were stained with anti-human CD274 PE (20 μL; BD Biosciences, 557924, RRID: AB_647198) or anti-human CD274 PE/cyanine 7 (5 μL; BioLegend, 374505, RRID: AB_2734433) according to the manufacturer’s instructions. IgG1 PE isotype antibody (1–20 μL; BD Biosciences, 349043, RRID: AB_400398) was used as an isotype control. For detection of PD-L2, anti-human CD273 antigen-presenting cells (APC; 20 μL; BD Biosciences, 557926, RRID: AB_647162) was used. Tumor HER2 expression was assessed using anti-HER-2/neu PE (20 μL; BD Biosciences, 340552, RRID: AB_400055). To study the expression dynamics in the presence of effector cytokines, tumor cells were incubated with exogenous IFN-γ (10 ng/mL; BioLegend, 570206) or HER2-CART for ∼24 hours prior to flow cytometry assessment. For detection of HER2-CAR expression, T cells were incubated with recombinant human HER2-Fc chimera protein (5 μL; R&D Systems, 1129-ER-050) followed by staining with goat anti-human IgG Fc PE (1 μL; Thermo Fisher Scientific, 12-4998, RRID: AB_465926) or goat anti-human IgG Fc FITC (5 μL; Millipore, AP113F, RRID: AB_92441) as previously described (34, 37). T-cell surface PD-1 was assessed using mouse anti-human CD279 APCs (5 μL; BD Biosciences, 558694, RRID: AB_1645458). Although inclusion of the 2A sequence leads to proportionate expression of both molecules, we assessed CPR expression in comparison with PD-1 detected on CAR and nontransduced (NT) T cells maintained under identical culture conditions and duration. We also confirmed the CPR coexpression by detection of the fluorescent mEmerald-GFP tag incorporated. For evaluation of T-cell phenotype and immune checkpoint receptor expression, autologous cocultures were set up (50,000 T cells to 100,000 tumor cells), and T cells were stimulated with the same number of fresh tumor cells at 48 and 96 hours and harvested for analysis. For evaluation of surface immunophenotype, T cells (containing both CD8^+^ and CD4^+^) were stained first for HER2-CAR using the method described above and then stained with mouse anti-human CD8 PerCP (5 μL; BD Biosciences, 347314, RRID: AB_400280), mouse anti-human CD4 FITC (5 μL; BD Biosciences, 340133, RRID: AB_400007), mouse anti-human CD45RA APCs (20 μL; BD Biosciences, 550855, RRID: AB_398468), or mouse anti-human CD197 (CCR7) PE (20 μL; BD Biosciences, 560765, RRID: AB_2033949). For detection of immune checkpoint receptors, T cells at baseline and after coculture with tumors were stained for HER2-CAR, followed by PD-1 APCs (as above), mouse anti-human LAG3 FITC (2 μL; Thermo Fisher Scientific, 11-2239-42, RRID: AB_2572486), or mouse anti-human CD366 (TIM3) APCs (2 μL; BioLegend, 345012, RRID: AB_2561718). As the sequence of the CPR extracellular domain was unmodified from native PD-1, log-change from baseline was used to compare the expression of immune checkpoint markers after stimulation. The log-change was calculated by first computing the log_10_ for each value using Microsoft Excel (RRID: SCR_016137). Then, each log value from after coculture time points was divided by the corresponding baseline value. The baseline surface phenotype of CARζ/CPR41BB and CAR41BBζ cells from healthy donors was assessed by staining with mouse anti-human CD8 PE Cy7 (2 μL; BD Biosciences, 560917, RRID: AB_2033970), mouse anti-human CD4 AmCyan A (2 μL; BD Biosciences, 562970, RRID: AB_2744424), mouse anti-human CD45RA BV421 (2 μL; BD Biosciences, 562885, RRID:AB_2737864), and mouse anti-human CCR7 Alexa Fluor 647 (2 μL; BD Biosciences, 560816, RRID: AB_2033948). To assess proliferative capacity, T cells were labelled with the cell proliferation dye eFluor 670 (Thermo Fisher Scientific) according to the manufacturer’s instructions and incubated with tumor cells at a 1:2 ratio in tissue culture–coated 24-well plates. Flow cytometry was performed at baseline and on day +3 of coculture. The cells were stained using mouse anti-human CD3 PE (5 μL; BD Biosciences, 347347, RRID: AB_400287) and analyzed using Accuri C6 (BD Biosciences, RRID: SCR_019591) or FACSCanto-II (BD Biosciences, RRID: SCR_018056).

### Imaging flow cytometry

T cells (2 × 10^6^ per condition) were cocultured with wild-type (WT) or PD-L1 KO LN229-GBM cells (1:1 ratio) in DMEM with 10% FCS and 1× GlutaMAX. Cells were collected and fixed with Cytofix (BD Biosciences) followed by BD Phosflow Perm/Wash Buffer (BD Biosciences). Cells were then stained with recombinant human ErbB2/HER2-Fc (0.5 μg; R&D Systems; 1129-ER-050) followed by goat anti-human IgG PE (0.5 μg; Thermo Fisher Scientific, 12-4998-82, RRID: AB_465926), DAPI (20 μg; MilliporeSigma; D9542-10MG), mouse anti-human CD279/PD-1 PE Cy7 (5 μL; BioLegend; 329918, RRID: AB_2159324), and phalloidin conjugated to Alexa Fluor 647 (1 μL; Thermo Fisher Scientific; A22287) and resuspended in 50 to 100 μL of permeabilization buffer. Samples were acquired using Amnis ImageStream Mk II (INSPIRE v200.1.388.0., Luminex, Luminex Amnis ImageStreamX Mk II System, RRID:SCR_018589), with a 60× objective, and ≥20,000 events (gated as RMS > 45 or “objects in focus”) were acquired using ISX. Compensation datasets were acquired in similar illumination conditions using single stain samples. Data was analyzed using IDEAS v6.2 (Luminex).

### ELISA and multiplex cytokine analysis

T cells (50,000–100,000 cells per well) were stimulated over 24 hours using HER2-Fc chimera (0–2 μg/mL; R&D Systems) and PD-L1–Fc chimera (0–5 μg/mL; R&D Systems) proteins immobilized in non–tissue culture–treated 96-well polystyrene plates (Thermo Fisher Scientific). The supernatant was analyzed using ELISA (Quantikine ELISA kits, R&D Systems) to quantify IL-2 and IFN-γ release. Supernatants collected at 24 hours from the T-cell (50,000-100,000) cocultures with GBM cells (100,000) were analyzed using ELISA as above or by using the Milliplex Map human cytokine/chemokine magnetic bead panel (MilliporeSigma) for IFN-γ, IL-2, TNF-α, GM-CSF, RANTES, and MIP-1α.

### Evaluation of T-cell cytotoxicity

Long-term tumor cell–killing kinetics of T cells was assessed using xCELLigence (Agilent) as described (37). Briefly, primary GBM cells and cell lines (10,000 cells) were allowed to proliferate in a microtiter plate and then treated with 1,000 T cells at 20 to 60 hours (T-cell to tumor cell ratio of ≥1–10). Tumor cell viability was monitored in real-time for 40 to 140 hours after the addition of T cells. The tumor lysis was indicated by the decreasing cell index. Assessment of short-term cytotoxicity of T cells after repeat antigen stimulation was done using ^51^Cr-release assay; T cells were incubated in a 96-well polystyrene plate coated with PD-L1–Fc (10 μg/mL) and HER2-Fc proteins (1 μg/mL) without exogenous cytokines for 7 days and then assessed for their antitumor activity against HER2^+^/PD-L1^+^ GBM cells. To evaluate the CARζ/CPR41BB cell ability to withstand repeat antigen stimulation and to exclude PD-L1/PD-L2–mediated off-target cytotoxicity, we used Incucyte Live-Cell Analysis (Sartorius). For serial cytotoxicity assessment, eGFP-expressing LN229-GBM (50,000 cells) were cocultured with T cells (10,000). For tumor rechallenge, on day 3, T cells were harvested, counted, and added to the plate prepared with fresh tumor cells (1:5 effector to target ratio). For confirming antigen specificity, human monocytic leukemia-derived eGFP-labeled THP1 cells (50,000), which lack HER2 but express PD-L1/PD-L2, were incubated with CARζ/CPR41BB cells (10,000) and examined for evidence of target lysis over 6 days. Triton-X was used as a positive control for cell lysis.

### Western blot

T cells (2 × 10^6^ per condition) at baseline and after coculture with LN229-GBM cells (T cells: tumor cell ratio of 1:1) were harvested and lysed with RIPA buffer (Cell Signaling Technology), including protease inhibitor cocktail (MilliporeSigma) and phosphatase inhibitor cocktail set III (MilliporeSigma). Protein was collected and quantified by Pierce BCA Protein Assay (Thermo Fisher Scientific), and samples were prepared at equal protein concentration for all samples using Laemmli sample buffer (Bio-Rad). Samples were run on precast 12% electrophoresis gels (Bio-Rad) and transferred to PVDF transfer stack membranes via iBlot2 (Thermo Fisher Scientific). The membranes were blocked with 5% milk and probed with mouse anti-human CD3ζ (Santa Cruz Biotechnology; sc-1239, RRID: AB_627020), mouse anti-human phosphoCD3ζ (BD Biosciences; 558402, RRID: AB_647307), and GAPDH (Cell Signaling Technology, 2118, RRID: AB_561053) mAbs followed by appropriate IRdye secondary antibodies (Li-Cor). Images were obtained by Li-Cor Odyssey CLx (RRID: SCR_014579) and ImageStudio 5.2 (RRID: SCR_015795). Band quantification was performed using Fiji (RRID: SCR_002285)/ImageJ (RRID: SCR_003070). CD3ζ and phosphoCD3ζ expressions were normalized between samples at each time point by GAPDH expression and expressed as a ratio, relative to the phosphoCD3ζ: CD3ζ at baseline in CARζ cells.

### Assessment of metabolomic profiles

Metabolomic profiles of CART were assessed using the Seahorse XFe96 Analyzer (RRID: SCR_019545; BCM Metabolomics Core, RRID: SCR_026214). Seahorse XF Cell Mito Stress Test Kit (Agilent, 103015-100) and Seahorse XF Cell Glycolysis Stress Test Kit (Agilent, 103020-100) were used to assess mitochondrial function and glycolysis, respectively. Mitochondrial function was assessed in resting CART (maintained in media with IL-7/IL-15) and after 7 days of continued stimulation with Fc-conjugated HER2 protein (1 μg/mL) ± PD-L1Fc (5 μg/mL). For short-term assessment of the oxygen consumption rate (OCR), CART cells were cocultured with WT LN229-GBM cells at a 1:2 T cells: tumor cell ratio. At 48 hours, CD8^+^ T cells were positively selected using CD8 MicroBeads, human (Miltenyi Biotec, 130-045-201). The extracellular acidification rate (ECAR) was assessed in resting cells and after 7 days of repeated stimulation with HER2Fc (1 μg/mL) and PD-L1–Fc (5 μg/mL). All T-cell samples were prepared for analysis following the manufacturer’s guidelines. Briefly, wells in Agilent Seahorse cell culture microplates (96-well) were coated with Cell-Tak and incubated for 1 hour at room temperature. T cells were resuspended in XF assay medium (Agilent, 103575-100) prepared containing 10 mmol/L glucose (Agilent, 103577-100), 2 mmol/L L-glutamine (Agilent, 103579-100), and 1 mmol/L pyruvate (Agilent, 103578-100) for Mito Stress Test Kit and 2 mmol/L L-glutamine for Glycolysis Stress Test Kit. T cells were seeded at 2 × 10^5^ cells per well for all assays, except for CD8^+^ T cells which were seeded at 1.5 × 10^5^ cells per well. The microplate was centrifuged at 200 × *g* for 2 minutes and transferred to a 37°C incubator (CO_2_-free) for 25 to 30 minutes to ensure cells were completely attached. The instrument was calibrated, and the OCR and ECAR were measured per the manufacturer’s instructions without modifications. All data were analyzed using Wave v2.6.0 (Agilent; RRID: SCR_024491) and Seahorse Report Generators (Agilent, RRID: SCR_019543). For assessment of mitochondrial reserve, the spare respiratory capacity (SRC) was calculated as the absolute difference between the basal and maximal respiration values after uncoupling. Both the SRC and SRC% (internally normalized relative to basal respiration) were generated using Seahorse XF Cell Mito Stress Test Report Generators (RRID: SCR_026230) without modifications.

### *In vivo* studies in orthotopic models

All animal studies were conducted according to research protocols approved by the BCM Institutional Animal Care and Use Committee, with adequate analgesia or general anesthesia or both as appropriate for the procedure performed. Application of heating pads was used during and after surgery to reduce hypothermia and facilitate recovery of the mice. Tumor engraftment was confirmed using *in vivo* imaging system (Xenogen IVIS 100 Imaging System, RRID: SCR_020901). Animals with failed tumor engraftment or significant welfare issues during anesthesia for tumor or T-cell injection were excluded.

#### Orthotopic GBM model

GBM xenografts were established in 12- to 13-week-old SCID mice (IcrTac- *Prkdc*^*scid*^, Taconic Biosciences, RRID: IMSR_TAC:ICRSC) by stereotactic injection of eGFP.Ffluc-expressing U373 (50,000 cells/mice; refs. 37, 39). For functional screening of CPR28 and CPR41BB in the U373-GBM model, T-cell treatment consisted of a single intratumoral injection of 1 × 10^6^ T cells (*n* = 5 mice/group) into the same stereotactic coordinates (39) on day +5 after the tumor inoculation. For assessment of antitumor activity and the treatment effect on survival without tumor progression using LN229-GBM xenografts, tumors were established by intracranial injection of eGFP.Ffluc-expressing LN229-GBM (50,000 cells/mice) cells into the right frontal cortex of NOD/SCID gamma (NSG) mice (NOD.Cg-*Prkdc*^*scid*^*Il2rg*^*tm1Wjl*^/SzJ; The Jackson Laboratory, RRID: IMSR_JAX:005557) as previously described (40). Mice with progressive GBM were randomized on day +11 to receive no treatment (*n* = 3) or the following treatment: CARζ/CPR41BB (*n* = 7), CARζ/CPR28 (*n* = 7), CAR41BBζ (*n* = 5), CAR28ζ (*n* = 5), CARζ (*n* = 5), and NT (*n* = 6) T cells. T-cell treatment consisted of intratumoral injections of 1 × 10^6^ CAR^+^ T cells on day +13 and 2 × 10^6^ CAR^+^ T cells on day +21. The mice were assessed for tumor regression or progression using serial bioluminescence imaging (BLI; 1–2 times/week for the first 4 weeks after tumor injection and once every 1–2 weeks thereafter). In both studies, mice were examined regularly for general well-being, neurologic deficits, and euthanasia endpoints. Progression-free survival (PFS) was defined by the duration of survival without tumor recurrence or progression, which was determined by a one-log increase in tumor volume, confirmed by repeated BLI at two consecutive time points.

#### Metastatic osteosarcoma model

Osteosarcoma xenografts were established in 5- to 6-week-old NSG mice (NOD.Cg-*Prkdc*^*scid*^*Il2rg*^*tm1Wjl*^/SzJ; The Jackson Laboratory, RRID: IMSR_JAX:005557) by intratibial injection of eGFP.Ffluc-expressing 143B tumor cells (50,000–250,000 cells/mice) suspended in 20 μL of PBS (Sigma-Aldrich). Serial BLI (Xenogen IVIS 100 Imaging System, RRID: SCR_020901) was used to assess tumor growth kinetics and development of lung metastasis. For assessment of immune effector–mediated systemic adverse effects, mice with established xenografts were randomized to CARζ/CPR41BB (*n* = 7) or CAR41BBζ (*n* = 5) groups and treated with relatively large doses of CART; 10 × 10^6^ T-cell per dose (suspended in 100 μL PBS), each administered intraperitoneally on day +5 and day +13 after tumor inoculation. After assessing the lung metastatic disease by BLI, mice were euthanized at various posttreatment time points using institutional guidelines; primary tumors and lung nodules were harvested for examination by hematoxylin and eosin staining, human CD3 (Dako, A0452, 1:50 dilution), and for PD-L1 (rabbit anti-human PD-L1; Cell Signaling Technology; 13684) immunohistochemistry (IHC). In an independent experiment to assess antitumor activity, mice with confirmed disease progression were randomized to receive no treatment (*n* = 5) or treatment with CARζ/CPR41BB (*n* = 5) or CAR41BBζ (*n* = 3) cells. On day +5 after tumor inoculation, mice were treated with a single intraperitoneal injection of 5 × 10^6^ T cells and assessed for overall survival at 30 days. In both *in vivo* studies, mice were followed with physical exams for general well-being, lower extremity deficits, weight loss, and other euthanasia endpoints.

### Statistical analysis

The Student two-tailed *t* test or Mann–Whitney test was used for comparison of two groups and one-way or two-way ANOVA with Tukey *post hoc* test for multiple comparisons, unless specified otherwise. For tumor volume assessments in mice, the signal intensity measured by BLI was log-transformed and summarized overtime. Survival analysis was done using the Kaplan–Meier method, and the differences were determined using the log-rank (Mantel–Cox) test (Holm–Sidak method as applicable). Significance was defined by *P* value of <0.05 unless specified otherwise. The HR was calculated using the Mantel–Haenszel method. All data are displayed as the mean ± SD unless specified otherwise. FlowJo v.10 (RRID: SCR_008520) was used for analysis of flow data, and ≥10,000 events were analyzed in each data set. Single-cell data from ImageStream microscopy were analyzed using R Project for Statistical Computing (RRID: SCR_001905). Histogram and Q–Q plots were used to qualitatively assess data normality and to visualize the data distribution. The IQR method was used to remove outliers, applying separately to each CAR design and its corresponding raw data. The Shapiro–Wilk test was used to assess normality for datasets with fewer than 5,000 inputs, whereas the Anderson–Darling test was applied to datasets with 5,000 or more inputs. Violin plots were generated illustrate the overall distribution and central tendency of each dataset. Due to nonnormal data distribution, the Mann–Whitney U test with Holm correction for multiple pairwise comparisons was used to assess statistical significance between groups. The relationship between two groups was evaluated using the Spearman rank correlation test. R packages used include ggplot2 (RRID: SCR_014601), dplyr (RRID: SCR_016708), tidyr (RRID: SCR_017102), and reshape2 (RRID: SCR_022679). All other data were analyzed using GraphPad Prism 9.0 (RRID: SCR_002798) or Microsoft Excel 2019 (RRID: SCR_016137).

### Data availability

All data are available within the article, supplementary information, and source files or from the corresponding author upon reasonable request. Data containing protected health information have been deidentified.

## Results

### GBM tumors show dynamic expression of PD-L1 and recruit PD-1 to the CAR immune synapse

**Figure 1:**
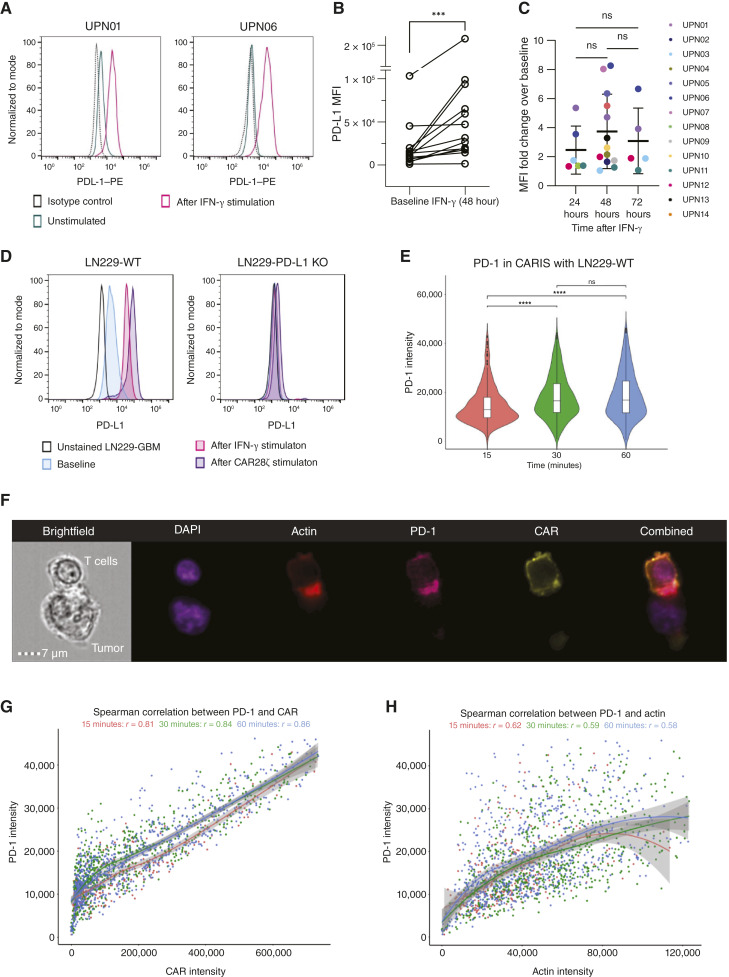
PD-L1 expression in GBM and PD-1 recruitment to the CARIS with GBM. **A,** Constitutive (UPN01) and inducible (UPN06) surface expression of PD-L1 in primary GBM cells after 24–48 hours of IFN-γ exposure. Representative results from two samples shown. UPN, unique patient number. **B,** PD-L1 median fluorescent intensity (MFI) on primary GBM (*n* = 14) before and at 48 hours of IFN-γ (10 ng/mL) exposure; ***, *P* < 0.001, Wilcoxon signed-rank test. **C,** Fold-change in PD-L1 MFI in GBM cells from baseline, at 24, 48, and 72 hours. Each color represents a single patient donor, with some measured at multiple time points. Data are shown as individual values with the mean ± SD. **D,** PD-L1 expression in WT LN229-GBM cells and LN229 with PD-L1 deletion (LN229-PD-L1 KO) at baseline and at 24 hours of exposure to IFN-γ (10 ng/mL) or CAR28ζ T cells. **E,** MFI of PD-1 in the immune synapse between CARζ cells and WT LN229-GBM cells at 15, 30, and 60 minutes (****, *P* < 0.0001; ns, *P* > 0.5, Kruskal–Wallis and Wilcox pairwise); ≥20,000 events were captured, and 300–1,000 CAR^+^ conjugates were examined for PD-1 recruitment to CARIS. The white box represents the IQR with horizontal lines at 25%, 50%, and 75%. **F,** Representative image capture showing the CARIS with tumor cell (WT LN229-GBM) and other parameters evaluated. Gating strategy is shown in the Supplementary material. **G,** Spearman correlation between PD-1 intensity and CAR intensity in the immune synapse at 15, 30, and 60 minutes. **H,** Spearman correlation between PD-1 intensity and actin intensity in the CARIS at 15, 30, and 60 minutes. ns, not significant.

The majority of GBMs exhibit cell surface expression of PD-L1, independent of their molecular characteristics (41–44). To study PD-L1 expression dynamics in the context of CART therapy, we examined tumor cells collected at the time of surgical resection from 14 patients with newly diagnosed or recurrent GBM at baseline and after exposure to the T-cell effector cytokine IFN-γ. When assessed using flow cytometry, 11 of 14 (79%) tumors showed detectable PD-L1 at baseline (Fig. 1A; Supplementary Fig. S1A and S1B). Exposure to exogenous IFN-γ (10 ng/mL) resulted in sustained or increased PD-L1 expression (Fig. 1A and B) in 13 of 14 (93%) tumors upon longitudinal assessment for up to 72 hours (Fig. 1C). Similarly, we observed PD-L1 upregulation in the HER2^+^ GBM cell lines LN229 (Supplementary Fig. S1C) and U373 (37) following treatment with IFN-γ or CART (Fig. 1D; Supplementary Fig. S1D). After antigen encounter, patient-derived CAR28ζ cells showed an increase in PD-1 expression after 48 hours of coculture with autologous HER2^+^ GBM cells (Supplementary Fig. S1E). PD-1 increases in activated T cells within 24 hours, and its recruitment to the immune synapse following TCR engagement with target cells mediates inhibitory signaling (39, 45–47). However, CART form a nonclassic immune synapse with tumor targets (48). To ascertain the relevance of the PD-1/PD-L1 axis independent of the type of costimulation, we studied the CAR immune synapse (CARIS) between first-generation HER2-CART (CARζ) and HER2^+^ LN229-GBM cells. As the conjugate formation between primary T cells and tumor cells is nonhomogenous, we examined ≥20,000 events by imaging flow cytometry (Amnis ImageStream) to allow for high-throughput screening of CARIS attributes. We observed increased intensity of endogenous PD-1 at the CARIS between CARζ and LN229-GBM from 15 to 30 minutes of cell conjugation (*P* < 0.0001, Kruskal–Wallis and Wilcox pairwise; Fig. 1E and F; Supplementary Fig. S2A–S2I), with no measurable differences between 30 and 60 minutes (*P* > 0.5, Kruskal–Wallis and Wilcox pairwise). The intensity of PD-1 correlated highly with that of CAR, and the correlation increased over time (r = 0.86 at 60 minutes, Spearman correlation), indicating CAR-dependent recruitment of PD-1 to the immune synapse (Fig. 1G). The correlation between actin, a signature of CARIS maturity, and PD-1 was relatively lower and decreased over time (Fig. 1H; r = 0.58 at 60 minutes, Spearman correlation). Together, these data suggest that upregulation of PD-L1 and recruitment of PD-1 to the CARIS could impair T-cell activation.

### Nondimerizing PD-1 CPR28 confers a functional advantage to CAR28ζ T cells *in vitro*

**Figure 2:**
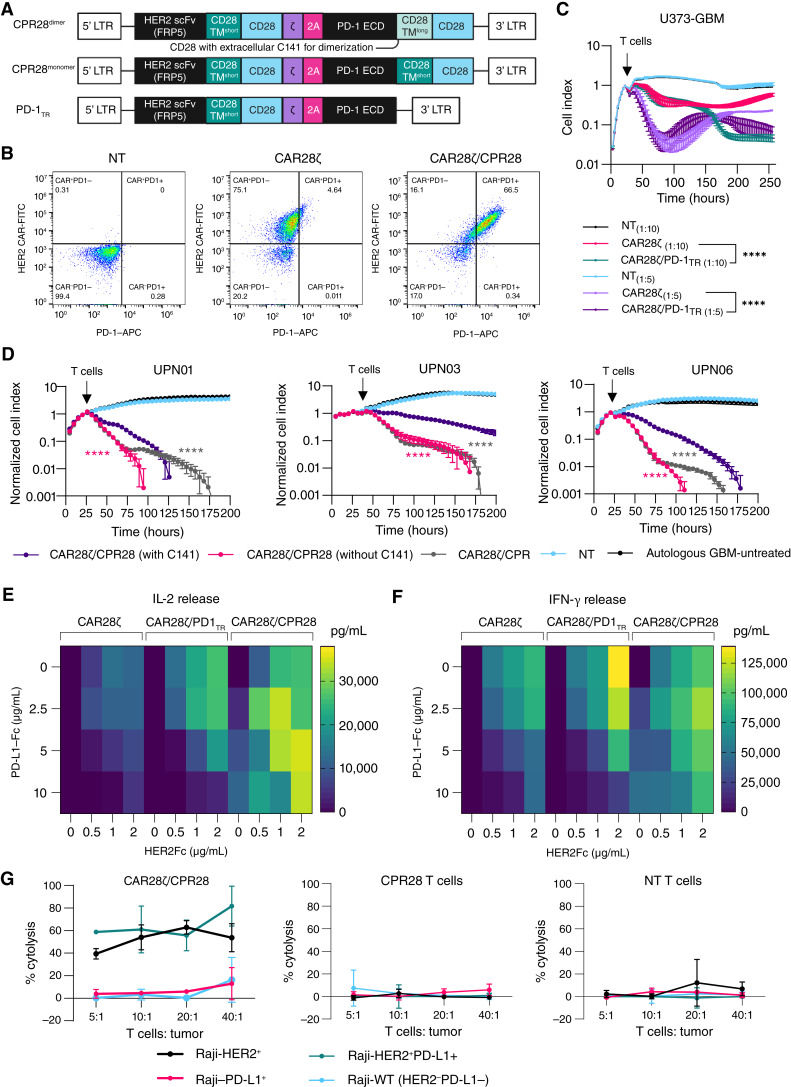
Design and functional screening of PD-1_TR_ and CPR28 molecules. **A,** Schematic representation of the bicistronic vectors encoding for the HER2-CAR with truncated PD-1 (PD-1_TR_) or CPR28. The CPR28^dimer^ included a membrane proximal extracellular cysteine residue (C141) required for CD28 homodimerization, as indicated. **B,** Flow cytometry analysis showing coexpression of HER2-CAR and PD-1 CPR on T cells. CAR28ζ and NT T cells from the same donor were used as controls for assessment of CPR expression determined by surface PD-1 detection. **C,** Long-term cytolytic function of CAR28ζ/PD-1_TR_, compared with CAR28ζ cells, against U373-GBM cells at effector to target ratios of 1:5 and 1:10 assessed using a cell-impedance based assay (xCELLigence). **D,** Comparison of the cytolytic ability of patient-derived CAR28ζ cells (*n* = 3) coexpressing CPR28^dimer^ or CPR28^monomer^ against autologous HER2^+^ GBM cells at an effector to target ratio of 1:10 by assessment of tumor cell viability in an xCELLigence assay. In **C** and **D**, error bars represent the mean ± SD at each time point. ****, *P* < 0.0001, two-way ANOVA with the Tukey multiple comparisons test. **E,** IL-2 and (**F**) IFN-γ release by CAR28ζ, CAR28ζ/PD-1_TR_, and CAR28ζ/CPR28 cells (100,000 T cells/well) upon stimulation with Fc-conjugated HER2 (0–2 μg/mL) and PD-L1 (0–5 μg/mL) proteins. Median values from a representative donor shown. **G,** CAR28ζ/CPR28 cell–induced lysis of Raji cells modified to express HER2 or HER2 and PD-L1 across different T-cell to tumor cell ratios but not the WT Raji cells (HER2^−^/PD-L1^−^) or those expressing PD-L1 alone in a ^51^Cr-release assay. T cells with CPR28 alone had no cytotoxic effect against HER2^+^ or PD-L1^+^ Raji cells, like NT T cells from the same donor. **A,** Created in BioRender. Navai, S. (2024) BioRender.com/l86h941.

PD-1, upon binding its ligand PD-L1, is shown to preferentially inactivate CD28 signaling in T cells (7). Therefore, to transform the inhibitory signal upon binding of PD-1 to its ligands, PD-L1 or PD-L2, within the TME, we engineered a synthetic CPR molecule by fusing the native PD-1 ectodomain with the CD28 transmembrane and endodomain (CPR28). Whereas CD28 exists as a disulfide-linked homodimeric glycoprotein (18), PD-1 is monomeric (44). We therefore designed two candidate CPR28 molecules with (CPR28^dimer^) and without (CPR28^monomer^) the inclusion of a sequence containing a membrane proximal extracellular cysteine residue (C141; refs. 47, 49, 50) that allows for dimerization. A synthetic receptor consisting of the PD-1 ectodomain, transmembrane domain, and endodomain truncated at AA222 before the immunoreceptor tyrosine-based switch motif was generated in parallel (PD-1_TR_). We used bicistronic transgenes (Fig. 2A) to coexpress PD-1_TR_ or CPR28 molecules with CAR28ζ in T cells (CAR28ζ/CPR28; Supplementary Fig. S3A) and confirmed their surface expression by flow cytometry (Fig. 2B; Supplementary Fig. S3B and S3C). In a long-term (>10 days) cytotoxicity assay against HER2^+^/PD-L1^+^ U373-GBM, PD-1 modulation with a nonsignaling PD-1_TR_ resulted in a markedly improved sustenance of CAR28ζ-mediated tumor lysis (*P* < 0.0001, two-way ANOVA with the Tukey test; Fig. 2C). When assessed using primary GBM cells (*n* = 3) with variable HER2 and PD-L1 expressions (Supplementary Fig. S3D), CAR28ζ cells expressing a CPR28^monomer^ demonstrated greater killing of autologous tumor cells compared with CPR28^dimer^ (*n* = 3; Fig. 2D). Tumor lysis induced by CAR28ζ/PD-1_TR_ was comparable with CAR28ζ/CPR28^monomer^ within the first 48 hours but slowed down subsequently in two of the three patient donors evaluated. We examined the effect of PD-1_TR_ and CPR28^monomer^ on cytokine production by activating CAR28ζ cells with plate-bound Fc-conjugated HER2 and PD-L1 proteins. PD-L1 dampened the production of the Th1 cytokines IL-2 (Fig. 2E) and IFN-γ (Fig. 2F) across CART, particularly at high concentrations, but this effect was partly overcome by increasing the target antigen density. Both CAR28ζ/PD-1_TR_ and CAR28ζ/CPR28^monomer^ showed higher IL-2 and IFN-γ release compared with CAR28ζ cells at various HER2 and PD-L1 protein densities. Nonetheless, the patterns of cytokine production and long-term cytotoxic function indicated that CPR-mediated costimulation had a beneficial effect. Therefore, we selected the CPR28^monomer^ design for further functional assessment. However, in this model system, exposure of CAR28ζ/CPR28 to PD-L1 resulted in low level, antigen-independent cytokine release. To definitively assess the effect of CAR or CPR engagement on T-cell specificity and cytolytic function, we force-expressed one or both molecules on HER2^−^/PD-L1^−^ Raji B lymphoblastic tumor cells (Supplementary Fig. S3E). In a 4-hour chromium release assay, CAR28ζ/CPR28 cells exhibited HER2-specific cytotoxicity against HER2^+^ Raji cells, whereas CPR28 cells did not induce lysis of PD-L1^+^ Raji cells (Fig. 2G), thus affirming that presence of the CAR target antigen is required for CAR28ζ/CPR28-mediated cell killing.

### Decoupling CAR T-cell activation signals attunes cytokine production while preserving cytotoxicity

**Figure 3:**
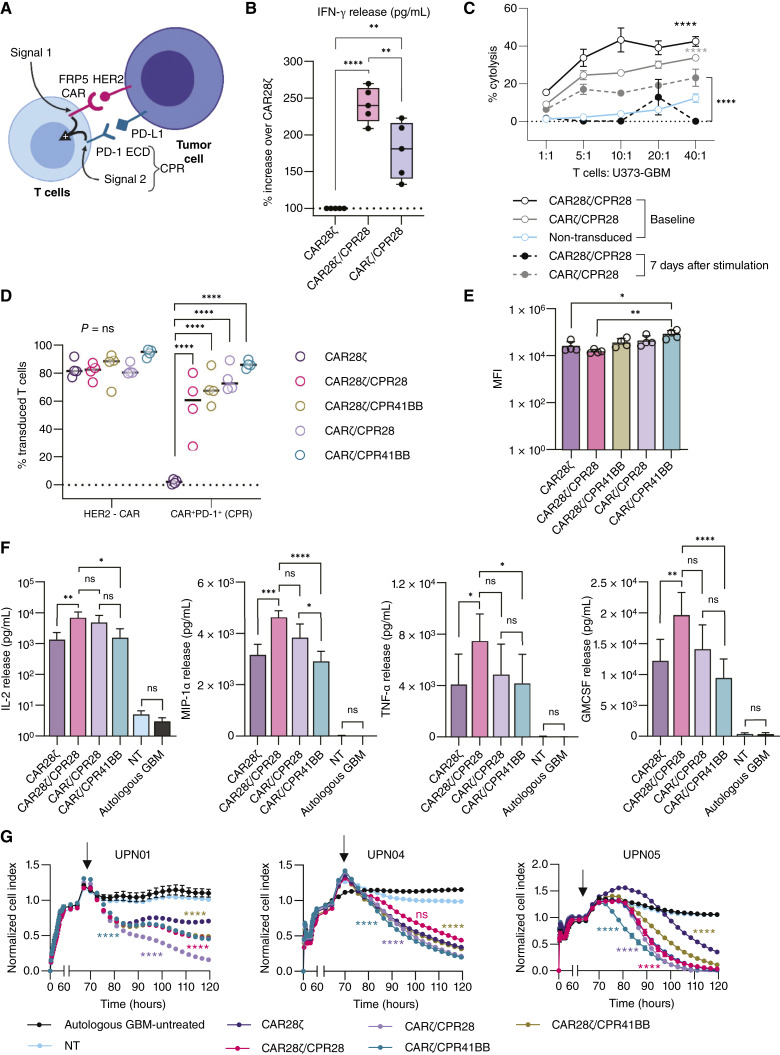
Effect of decoupling signal 2 from the CAR on T-cell function. **A,** Illustration depicting the bipartite T-cell activation through signal 1 delivery from CAR engaging the HER2 antigen and signal 2 from binding of CPR with PD-L1 (or PD-L2). **B,** Percent increase in IFN-γ production by CAR28ζ/CPR28 and CARζ/CPR28 compared with CAR28ζ cells (50,000 T cells/well) at 24 hours of coculture with autologous GBM cells (*n* = 5 patients). Effector (100,000 T cells) to target ratio of 1:1. **, *P* < 0.01; ****, *P* < 0.0001, one-way ANOVA with the Tukey multiple comparisons test. **C,** Cytolytic function of CAR28ζ/CPR28 and CARζ/CPR28 cells assessed using 4-hour ^51^Cr-release assay at baseline and at 7 days of persistent T-cell stimulation through the CAR. NT T cells had poor viability after prolonged stimulation without added homeostatic cytokines and were not evaluable. ****, *P* < 0.0001, two-way ANOVA with the Tukey multiple comparisons test. **D,** Percent of total T cells expressing HER2-CAR on cell surface, and percent of CAR^+^ T cells detected with PD-1 (surrogate marker for CPR), respectively, amongst different CPR/CART groups (*n* = 4 patients) using flow cytometry. **E,** Median fluorescent intensity (MFI) of HER2-CAR detected in transduced T cells shown in **D**. In **D** and **E**, only statistically significant differences are shown, *, *P* < 0.05; **, *P* < 0.01; ****, *P* < 0.0001, one-way ANOVA with the Tukey multiple comparisons test. **F,** Multiplex analysis for proinflammatory cytokines (IL-2, MIP-1α, TNF-α, and GM-CSF) in autologous T-cell and GBM coculture (*n* = 4 patients) supernatants at 24 hours. UPN, unique patient number. *, *P* < 0.05; **, *P* < 0.01; ****, *P* < 0.0001, two-way ANOVA with the Tukey multiple comparisons test. **G,** Assessment of long-term cytolytic function of CPR/CART against autologous GBM cells (*n* = 3 patients) using cell impedance–based xCELLigence assay. Statistical differences (denoted by the color key) shown are in comparison to control treatment CAR28ζ cells overtime. ****, *P* < 0.0001, two-way ANOVA with the Tukey multiple comparisons test. In **G,** black arrow indicates addition of T cells. **A,** Created in BioRender. Navai, S. (2019) BioRender.com/p40z816.

Although our primary intent was to enhance the CAR28ζ performance, excessive CD28 costimulation resulting from upregulation of the PD-1 and PD-L1/PD-L2 feedback loop could induce T-cell exhaustion, a dysfunctional state characterized by poor effector function (51, 52). Alternatively, providing signal 2, decoupled from the CAR, through a CPR molecule could recruit the necessary components for optimal T-cell activation (Fig. 3A) and simultaneously modulate the PD-1 axis while adapting effector function to both target antigen density and dynamic changes in PD-L1/PD-L2. Accordingly, we coexpressed a CPR28 molecule with a first-generation HER2-CAR (CARζ/CPR28; Supplementary Fig. S4A), eliminating the two-fold CD28 costimulation. Whereas T-cell proliferation and IL-2 production are highly sensitive to PD-1 upregulation, inhibition of IFN-γ production and cytotoxicity requires a stronger PD-1 signal (53). Therefore, we first measured IFN-γ, a key effector cytokine in mediating T-cell cytotoxicity, to assess the effect of signal splitting with CPR28. At 24 hours of coculture with autologous GBM, we observed a consistent pattern across all patient donors (*n* = 5), wherein CPR/CART demonstrated greater IFN-γ release over CAR28ζ, but with a marked difference between CARζ and CAR28ζ cells coexpressing CPR28 (one-way ANOVA with the Tukey test; Fig. 3B). We evaluated the effect of CPR28 on cytotoxic function of CARζ and CAR28ζ cells continuously activated (7 days) with plate-bound HER2 and PD-L1 proteins by using 4-hour chromium release assay. At baseline, CAR28ζ/CPR28 cells were the most effective against HER2^+^/PD-L1^+^ U373-GBM cells, but they exhibited the greatest decline in cytotoxic activity following chronic stimulation (two-way ANOVA with the Tukey test; Fig. 3C). Given the abundance of PD-L1 and PD-L2 in GBM (41, 42, 54–56) and their conditional overexpression in the presence IFN-γ, the type of costimulation received through CPR could impact the function and longevity of CART.

To examine how costimulatory domains affect bipartite CART activation, we developed a CPR molecule containing a 41BB endodomain and a CD8α transmembrane domain (CPR41BB; Supplementary Fig. S4B and S4C). We found that, similar to CPR28, CPR41BB promoted IL-2 release through PD-L1–mediated costimulation but, unlike CPR28, did not produce cytokine in the absence of HER2 (Supplementary Fig. S4D). Following repeat antigenic stimulation, CARζ/CPR41BB cells maintained their cytotoxic function against HER2^+^ LN229-GBM cells (Supplementary Fig. S4E). The cytotoxicity was antigen-dependent, as CARζ/CPR41BB cells did not lyse HER2^−^/PD-L1^+^/PD-L2^+^ human leukemia monocytic cells (THP1; Supplementary Fig. S4F and S4G). We used bicistronic vectors to create different CPR/CART variants based on our clinically tested HER2-CAR and generated control T cells in parallel, from both healthy donors (*n *= 5) and patients with GBM (*n *= 5). The proportional surface expression of HER2-CAR and PD-1 CPR within each donor was assessed using flow cytometry across CPR/CART groups (Fig. 3D and E; Two-way ANOVA with the Tukey test). We confirmed these results by incorporating an mEmerald-GFP tag in the CPR41BB molecule (Supplementary Fig. S4H). At 24 hours after coculture with autologous GBM cells, we observed substantial variation in cytokine release between patients (*n* = 4), but the pattern across CART conditions was consistent within each patient, with CAR28ζ/CPR28 cells demonstrating higher production of proinflammatory cytokines (IL-2, MIP-1α, TNF-α, and GM-CSF; two-way ANOVA with the Tukey test; Fig. 3F). CART from individual patients (*n* = 4) differed in their antitumor activity against autologous HER2^+^ GBM cells in long-term cytotoxicity assays (Fig. 3G). Across patients, CPR-expressing CART cells consistently demonstrated sustained tumor lysis, superior or comparable to control CAR28ζ cells, with improved cytolytic activity particularly evident in CARζ cells receiving costimulation through CPR28 or CPR41BB. Thus, bipartite T-cell activation through PD-1 CPR tempered the cytokine release while conserving or enhancing the *in vitro* antitumor function of CARζ cells.

### Bipartite activation of CART through CPR41BB costimulation is more favorable

**Figure 4:**
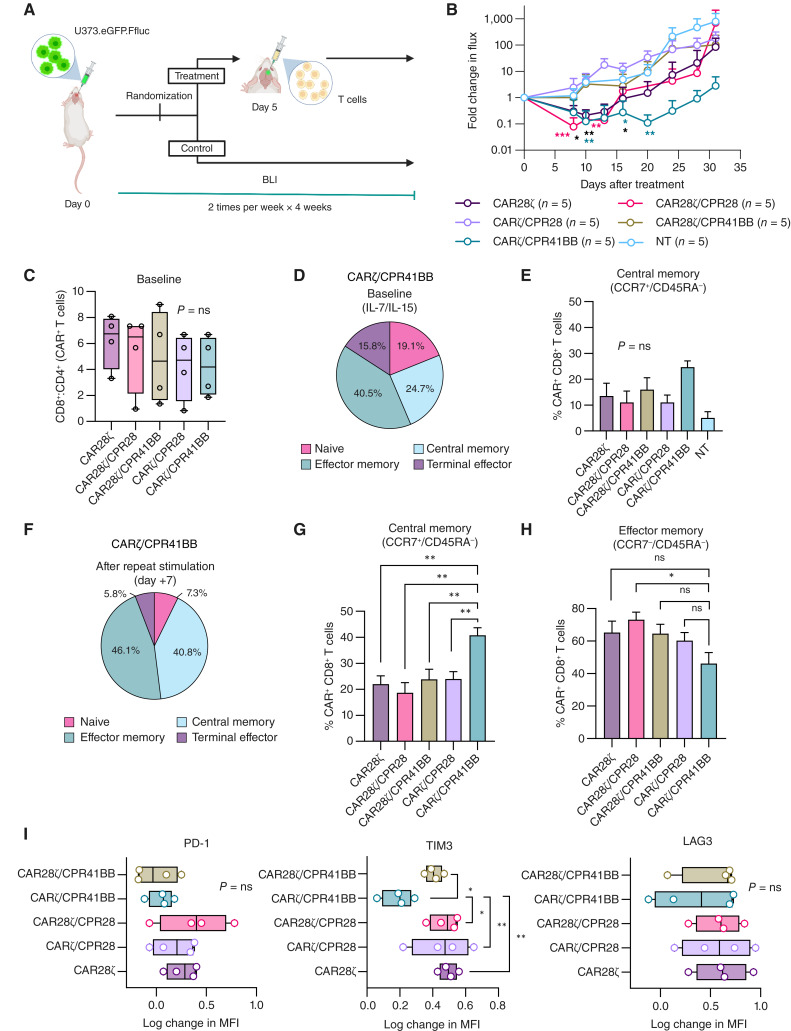
Phenotypic and functional profile of CART receiving bipartite activation signals through CPR41BB costimulation. **A,***In vivo* functional screening of CARζ and CAR28ζ cells coexpressing CPR28 or CPR41BB against orthotopic xenografts of HER2^+^PD-L1^+^ U373-GBM in SCID mice (*n* = 5 per group). **B,** Fold change in tumor burden after treatment relative to the tumor volume before intratumoral injection of T cells (day 0), quantified by serial BLI. *, *P* < 0.05; **, *P* < 0.01, two-way ANOVA with the Tukey multiple comparisons test. Data are shown as the mean ± SD. **C,** CD8^+^: CD4^+^ ratio in CAR-expressing T cells among CPR/CART from patients with GBM (*n* = 4) as assessed by flow cytometry. Box plots show minimum to maximum with individual values. ns, *P* > 0.5, one-way ANOVA with the Tukey multiple comparisons test. **D,** Pie graph demonstrating immunophenotype distribution of CARζ/CPR41BB cells (*n* = 4 patients) at baseline. Percentages shown represent the mean value. Immunophenotype is defined as follows: naïve, CCR7^+^/CD45RA^+^; central memory, CCR7^+^/CD45RA^−^; effector memory, CCR7^−^/CD45RA^−^; and terminal effector, CCR7^−^/CD45RA^+^. **E,** The percentage CAR^+^CD8^+^ central memory (CCR7^+^/CD45RA^−^) T cells did not significantly differ amongst different CPR/CART groups (*n* = 4 patients). The mean ± SEM is shown. ns, *P* > 0.5, one-way ANOVA with the Dunnett multiple comparisons test. **F,** Pie graph demonstrating immunophenotype distribution of CARζ/CPR41BB cells (*n* = 4 patients) after 7 days of continued stimulation by repeat cocultures with autologous GBM cells. Percentages shown represent the mean value. On day 7 of repeated stimulation, CARζ/CPR41BB cells (*n* = 4 patients) demonstrated (**G**) a significantly higher proportion of CAR^+^/CD8^+^ cells with the central memory phenotype compared with other CPR/CART groups and CAR28ζ, and (**H**) a significantly lower proportion of CAR^+^/CD8^+^ cells with the effector memory phenotype compared with CAR28ζ/CPR28 cells. The mean ± SEM is shown. *, *P* < 0.05; **, *P* < 0.01, one-way ANOVA with the Dunnett multiple comparisons test. **I,** Log-change in the median fluorescent intensity (MFI) of T-cell surface PD-1, LAG3, and TIM3 at 7 days of repeat coculture with autologous GBM cells (*n* = 4 patients) from baseline. Box plots show minimum to maximum with individual values. *, *P* < 0.05; **, *P* < 0.01, two-way ANOVA with the Tukey multiple comparisons test. ns, not significant.

First-generation CART cells have poor antitumor function *in vivo*, largely due to limited proliferative capacity and insufficient cytokine production (57, 58). Contrary to the robust CART proliferation and effector response elicited by CD28, 41BB costimulation favors central memory differentiation, enhances persistence, and has been associated with improved PFS in CD19-CART–treated patients (18, 59). To assess how CAR and CPR design impact the T-cell functional dynamics *in vivo*, we screened the candidate CPR/CART in an orthotopic xenograft model of U373-GBM (Fig. 4A), established as previously described (37, 39). Following a single intratumoral injection of 1 × 10^6^ T cells (*n* = 5 mice/group) on day +5 after tumor inoculation, CAR28ζ/CPR28 induced a rapid decrease in tumor volume compared with the pretreatment value (*, *P* < 0.05; **, *P* < 0.01, two-way ANOVA with the Tukey test; Fig. 4B). The kinetics of tumor regression immediately after treatment were comparable between CARζ/CPR41BB and CAR28ζ, but the response was better sustained in the CARζ/CPR41BB group. Whereas CAR28ζ/CPR41BB slowed tumor growth initially, the activity was not durable, suggesting that the potential synergy between costimulatory molecules did not provide an advantage.

To decipher the effect of CAR and CPR design on the T-cell immunophenotype, we examined the composition of CPR/CART before and after tumor encounter *in vitro*. All CART products generated from patients with GBM (*n* = 4) contained a higher proportion of CD8^+^ T cells (Supplementary Fig. S5A and S5B), with a comparable CD8^+^: CD4^+^ ratio in CAR-expressing T cells (*P* > 0.05, one-way ANOVA with the Tukey test; Fig. 4C). At baseline (expanded in media with IL-7/IL-15), both CAR28ζ and CPR/CART were mainly composed of central memory (CCR7^+^/CD45RA^−^) and effector memory (CCR7^−^/CD45RA^−^) subsets within CAR^+^CD8^+^ T cells (*P* = not significant; one-way ANOVA with the Dunnett test; Fig. 4D and E; Supplementary Fig. S5B). Following repeated stimulation over 7 days using autologous HER2^+^ GBM cells with constitutive or inducible PD-L1 expression, the CD8^+^:CD4^+^ ratio remained unchanged across T-cell conditions (*P* > 0.05, two-tailed Student *t* test; Supplementary Fig. S5C). CARζ/CPR41BB cells showed differential expansion of the central memory (CCR7^+^/CD45RA^−^) compartment after prolonged tumor encounter compared with all other CART cells (*, *P* < 0.05; **, *P* < 0.01, one-way ANOVA with the Dunnett test; Fig. 4F and G; Supplementary Fig. S5D), whereas the proportion of CAR^+^CD8^+^ effector memory T cells differed between CARζ/CPR41BB and CAR28ζ/CPR28 but not with others (*, *P* < 0.05, one-way ANOVA with the Dunnett test; Fig. 4H). We measured the surface expression of immune checkpoint receptors in CART (*n* = 4) after repeated challenge with autologous tumor cells and found significantly lower levels of TIM3 (*, *P* < 0.05; **, *P* < 0.01, two-way ANOVA with the Tukey test; Fig. 4I; Supplementary Fig. S5E and S5F) and a trend toward lower PD-1 in CARζ/CPR41BB cells. Collectively, CPR41BB costimulation conferred phenotypic characteristics associated with favorable outcomes after adoptive transfer to CARζ cells (51, 60–62).

### CARζ/CPR41BB cells exhibit distinct functional dynamics

**Figure 5:**
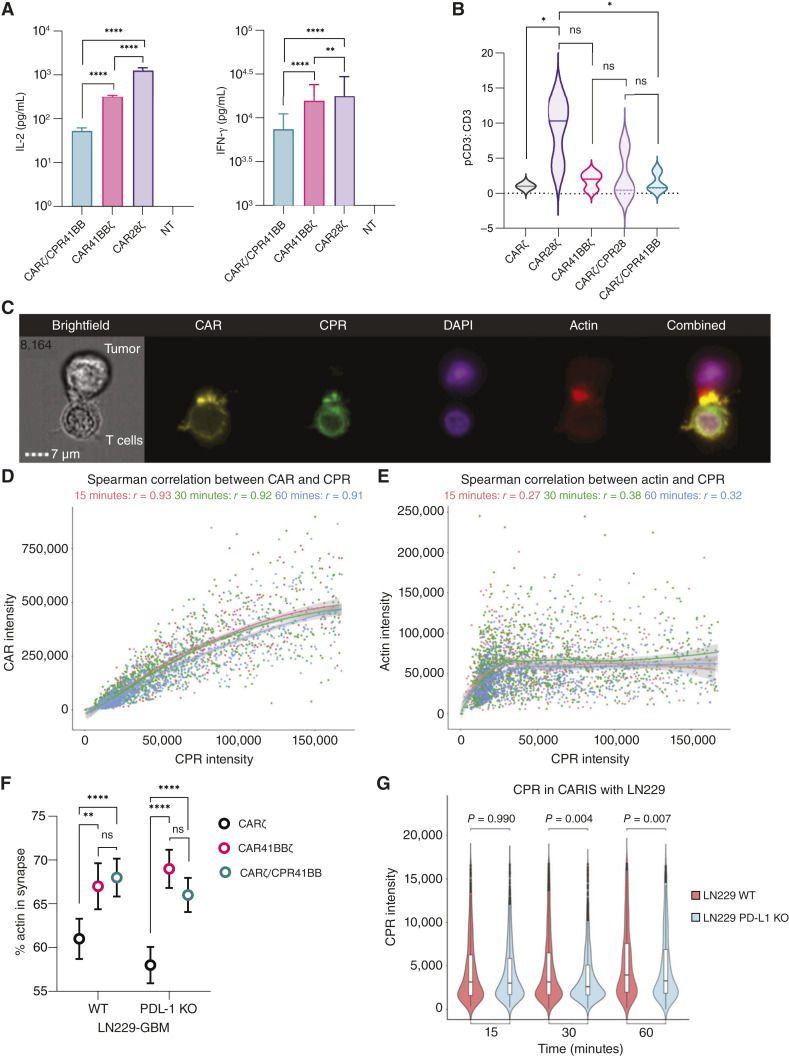
Dynamics of CARζ/CPR41BB T-cell activation and CARIS formation in comparison with CAR41BBζ cells. **A,** CARζ/CPR41BB cells (*n* = 3 donors) demonstrated significantly lower IL-2 and IFN-γ release compared with CAR41BBζ cells at 24 hours of coculture with LN229-GBM cells, with CAR28ζ cells consistently showing the higher Th1 cytokine production. Data are shown as the mean ± SD. **, *P* < 0.000; ****, *P* < 0.0001; two-way ANOVA with the Tukey multiple comparisons test. **B,** Western blot analysis for CAR-phosphoCD3ζ (pCD3) in T cells in a resting state (maintained in culture with IL-7/IL-15). The pCD3 to CD3 ratio is normalized to CARζ in each donor. *, *P* < 0.05; one-way ANOVA with the Tukey multiple comparisons test. **C,** Representative image capture showing the CARIS with tumor cell (LN229-GBM) and different CARζ/CPR41BB immune synapse parameters evaluated. Gating strategy is shown in the Supplementary Material. Spearman correlation (**D**) between the intensity of CAR and CPR at the CARIS and (**E**) between the intensity of actin and CPR at the CARIS, both assessed by imaging flow cytometry at 15, 30, and 60 minutes. **F,** CARζ/CPR41BB and CAR41BBζ cells show significantly higher percent (%) of F-actin at the CARIS compared with CARζ cells. ns, *P* > 0.5; **, *P* < 0.01; ***, *P* < 0.0001, two-way ANOVA with the Tukey *post hoc* test. Data are shown as the mean with 95% confidence interval (CI). **G,** CARζ/CPR41BB cells show significantly higher CPR intensity in the CARIS with WT LN229 GBM cells at 15, 30, and 60 minutes compared with conjugates with LN229-PD-L1 KO (Kruskal–Wallis and Wilcox pairwise). CPR intensity increased over time in both conditions. ns, not significant.

Costimulatory switch receptors have been shown to augment CART function (8). However, the understanding of T-cell activation and the subsequent downstream events when signal 2 is delivered via an independent ligand–receptor interaction, as opposed to the CAR ligation, is limited. Therefore, we compared phenotypic and functional properties of CARζ/CPR41BB cells with those of CAR41BBζ cells. At baseline, the proportion of CD8^+^ and CD4^+^ T cells and the immunophenotype of these subsets were similar between CARζ/CPR41BB and CAR41BBζ cells (*n* = 3 donors; Supplementary Fig. S6A and S6B). In long-term assessment of cytotoxicity, CARζ/CPR41BB cells were more efficient in inducing complete and sustained elimination of LN229-GBM cells over CAR41BBζ cells (****, *P* < 0.0001; two-way ANOVA with the Tukey test; Supplementary Fig. S6C). CARζ/CPR41BB cells (*n* = 6 donors) expanded comparably in media containing IL-7 and IL-15 (*P* = not significant; two-way ANOVA with the Tukey test; Supplementary Fig. S6D) and exhibited proliferative capacity similar to that of second-generation CART (*n* = 3 donors) when exposed to HER2^+^/PD-L1^+^ tumor cells for 72 hours (Supplementary Fig. S6E). The production of Th1 cytokines (IL-2 and IFN-γ) by CARζ/CPR41BB cells (*n* = 3) was lower at 24 hours after coculture with LN229-GBM cells compared with both CAR41BBζ and CAR28ζ (**, *P* < 0.000; ****, *P* < 0.0001; Two-way ANOVA with the Tukey test; Fig. 5A). Multiplex analysis of the coculture supernatant from two additional T-cell donors showed relatively lower secretion of proinflammatory cytokines (GM-CSF, IFN-γ, IL-2, TNF-α, RANTES, and MIP-1α) by CARζ/CPR41BB, with a pattern more similar to CARζ cells (Supplementary Fig. S6F).

CAR design and choice of costimulatory domain influence antigen-independent T-cell activation (63, 64) and CD3ζ phosphorylation dynamics (19, 65). We examined CAR-CD3ζ phosphorylation by Western blot and found that, at rest (in media with IL-7/IL-15), antigen-independent activation was significantly lower in CARζ/CPR41BB cells, whereas constitutive signaling was observed in CAR28ζ cells across all donors (*, *P* < 0.05, one-way ANOVA with the Tukey test; *n* = 3; Fig. 5B; Supplementary Fig. S6G). To evaluate the immunologic synapse, which is crucial for antigen-dependent T-cell activation, we studied the interface between CARζ/CPR41BB-mEmerald-GFP and LN229-GBM cells using imaging flow cytometry (*n* = 3 donors, Supplementary Fig. S2A–S2I). From cocultures containing equal numbers of targets and effectors across T-cell conditions, we examined >20,000 conjugates at 15, 30, and 60 minutes and assessed the dynamics of CPR41BB recruitment to the CARIS (Fig. 5C) with both WT (PD-L1^+^/PD-L2^+^) and PD-L1 KO (PD-L1^−^/PD-L2^+^) LN229-GBM cells (Supplementary Fig. S7A and S7B). In all conditions, the intensity of actin, a marker of mature immune synapse and a prerequisite for T-cell cytotoxic function, correlated with the recruitment of CAR (Supplementary Fig. S7C–S7E). In the CARIS with WT LN229-GBM, the intensity of CPR and CAR was strongly correlated at all time points, with the correlation pattern indicating higher CPR recruitment over time (r = 0.93 at 15 minutes, Spearman correlation; Fig. 5D). The recruitment of actin correlated with CPR at low intensities, but higher levels of CPR did not correspond to increased actin at the CARIS (r = 0.27 at 15 minutes, Spearman correlation; Fig. 5E). When compared across CART, F-actin polarization in CARζ/CPR41BB conjugates was higher compared with first-generation CARζ cells in both WT and PD-L1 KO LN229-GBM conditions by 60 minutes (**, *P* < 0.01; ****, *P* < 0.0001, two-way ANOVA with the Tukey test; Fig. 5F), whereas actin enrichment at the synapse interface was similar between CARζ/CPR41BB and second-generation CAR41BBζ cells (*P* = 0.826, two-way ANOVA with the Tukey test). In the CARIS with both WT and PD-L1 KO LN229-GBM, the recruitment of CPR increased over time, but the intensity was found to be dependent on the expression of PD-L1 on tumor targets (*P* = 0.99 at 15 minutes, *P* = 0.004 at 30 minutes, *P* = 0.007 at 60 minutes, Kruskal–Wallis and Wilcox pairwise; Fig. 5G). Functional testing against PD-L1 KO LN229-GBM cells confirmed that, independent of the intensity of CPR, CARζ/CPR41BB cells formed a cytotoxic immune synapse capable of triggering robust lysis of tumor targets (Supplementary Fig. S7F). Therefore, the functional characteristics of CARζ/CPR41BB cells were unique, showing activation dynamics that fell between those of CARζ and CAR41BBζ cells.

### Decoupling activation signals with CPR41BB maintains the favorable metabolism of 41BB costimulation

**Figure 6:**
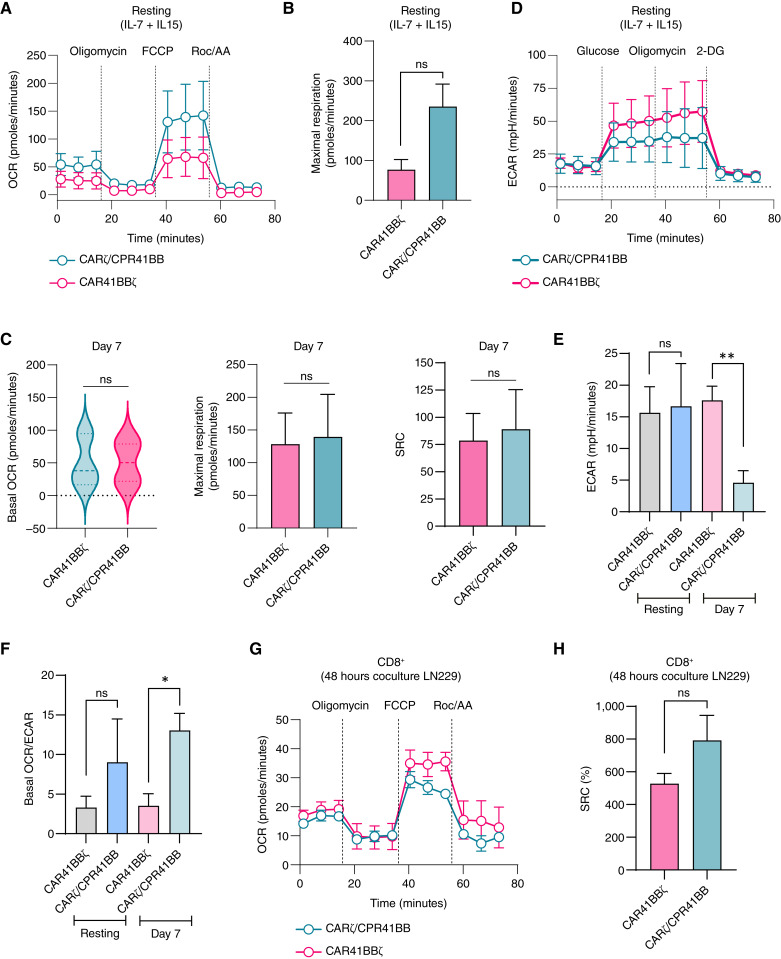
Metabolomic parameters of CARζ/CPR41BB cells in comparison with CAR41BBζ cells. **A,** OCR measurements of resting (cultured in media containing in IL-7 and IL-15) CARζ/CPR41BB and CAR41BBζ cells (*n* = 3 donors; 200,000 T cells per well) under basal metabolic conditions and after the addition of mitochondrial inhibitors. **B,** Comparison of maximal respiration between CARζ/CPR41BB cells and CAR41BBζ cells at baseline (day 0). *P* = 0.059, Student two-tailed *t* test. **C,** Basal OCR, maximal respiration, and SRC between CAR41BBζ and CARζ/CPR41BB cells after 7 days of stimulation with Fc-conjugated HER2 and PD-L1 proteins. ns, *P* > 0.05, Student two-tailed *t* test. **D,** The ECAR in resting CARζ/CPR41BB and CAR41BBζ cells. **E,** The ECAR of CARζ/CPR41BB and CAR41BBζ at baseline and at 7 days of continued stimulation with plate-bound HER2 and PD-L1 proteins. ns, *P* > 0.05; **, *P* < 0.01, Student two-tailed *t* test. **F,** Basal OCR to ECAR ratio in CARζ/CPR41BB compared with CAR41BBζ cells at rest and at 7 days of continued stimulation with HER2 and PD-L1 proteins. ns, *P* > 0.05; *, *P* < 0.05, Student two-tailed *t* test. **G,** The OCR in CD8^+^ CARζ/CPR41BB and CAR41BBζ cells (*n* = 3 donors; 150,000 T cells per well) following 48-hour coculture with LN229-GBM cells (effector to tumor ratio 1:2). **H,** The SRC of CD8^+^ CARζ/CPR41BB and CAR41BBζ cells after 48 hours of coculture with LN229 GBM cells. ns, *P* > 0.05, Student two-tailed *t* test. The OCR and ECAR measurements are shown as the mean ± SEM. Data shown denote the IQR in the violin plot and the mean ± SEM in the bar graphs. ns, not significant.

CAR signaling domains influence T-cell metabolism, with 41BB costimulation favoring non-glycolytic metabolism and CD28 costimulation promoting glycolysis and effector differentiation (66, 67). To investigate whether decoupling activation signals via CPR41BB impacted T-cell metabolism, we examined the metabolic profile of CARζ/CPR41BB and CAR41BBζ cells (*n* = 3 donors). Using live cell analysis, we assessed glycolysis and oxidative phosphorylation in resting cells (maintained in IL-7/IL-15) and after 7 days of continued stimulation with Fc-conjugated HER2 and PD-L1 proteins. We assessed mitochondrial function by measuring the OCR at baseline and after sequential additions of oligomycin (inhibitor of mitochondrial ATP synthase), carbonyl cyanide-p-trifluoromethoxyphenylhydrazone (uncoupling agent), and rotenone/actinomycin A (inhibition of complexes I and III). In resting cells, the OCR varied among donors (Fig. 6A; Supplementary Fig. S8A) but was similar between CART conditions, with CARζ/CPR41BB exhibiting a trend toward higher maximal respiration (*P* = 0.059, Student two-tailed *t* test; Fig. 6B). After 7 days of antigenic stimulation in the presence of PD-L1, the basal OCR, maximal respiration, and SRC were comparable between CARζ/CPR41BB and CAR41BBζ cells (*P* > 0.05, Student two-tailed *t* test; Fig. 6C). The OCR in CARζ/CPR41BB stimulated with HER2Fc alone varied from those receiving dual stimulation (Supplementary Fig. S8B). The ECAR, a measure of glucose utilization and lactic acid production, of resting CARζ/CPR41BB cells was similar to that of CAR41BBζ cells (*P* > 0.05, Student two-tailed *t* test; Fig. 6E). After stimulation, CARζ/CPR41BB cells demonstrated a significantly lower basal ECAR compared with CAR41BBζ cells (*P* = 0.003, Student two-tailed *t* test). Glycolysis was relatively lower in CARζ/CPR41BB cells at rest, but after antigenic stimulation, we observed a significant shift toward mitochondrial metabolism, shown by a much higher basal OCR to ECAR ratio at 7 days of repeated HER2 and PD-L1 exposure (*P* = 0.033, Student two-tailed *t* test; Fig. 6F). On the other hand, upon assessment of the OCR after short-term antigenic stimulation using WT LN229-GBM cells (Fig. 6G), we did not observe significant differences in the SRC of CD8^+^ T-cell, which were sorted from cocultures at 48 hours (*P* > 0.05, Student two-tailed *t* test; Fig. 6H). Thus, dissociating 41BB costimulation from the CAR preserved the favorable mitochondrial metabolism in CARζ/CPR41BB cells and may be advantageous following repeated antigenic exposure.

### CARζ/CPR41BB cells show enhanced *in vivo* antitumor activity against GBM after locoregional delivery

**Figure 7:**
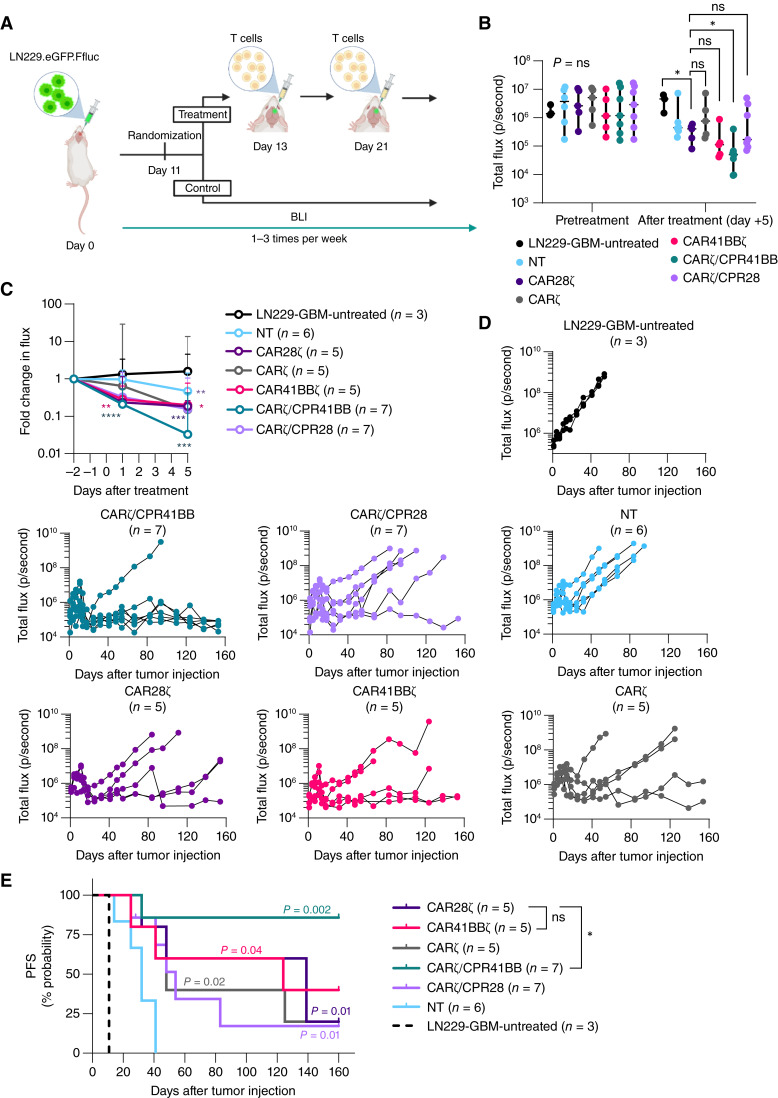
*In vivo* antitumor activity of locoregionally delivered CARζ/CPR41BB cells in an orthotopic GBM model. **A,** Experimental schema for *in vivo* functional testing of CARζ/CPR41BB cells in a LN229-GBM orthotopic xenograft model. Mice were injected with eGFP.FFluc-expressing tumor cells on day 0 and randomized to treatment groups on day 11. Treatment consisted of two intracranial T-cell injections on days 13 and 21. Tumors were monitored by BLI. **B,** Comparison of tumor volumes between treatment and control groups after randomization but prior to treatment and 5 days after the first T-cell injection as measured by serial BLI. The statistical differences relative to control treatment CAR28ζ are shown. **C,** Quantification of treatment response by fold change in tumor burden after T-cell injection (day 0 for posttreatment assessment) within each treatment group, and (**D**) differences in tumor control over time as determined by BLI over 160 days after tumor inoculation (day 0). **E,** Kaplan–Meier analysis at 160 days after tumor injection showing probability of PFS in mice treated with HER2-targeted CART with or without CPR coexpression compared with untreated mice or those receiving NT T cells; all CART-improved PFS (*P* value color denotes significance over NT cells). Ticks represent censored subjects. *, *P* < 0.05; **, *P* < 0.01; ***, *P* < 0.001; Mann–Whitney test for comparison between two groups; two-way ANOVA with the Tukey *post hoc* test for multiple comparisons. Log-rank test (Holm–Sidak) for survival analysis.

To comprehensively evaluate the function of CARζ/CPR41BB cells and to directly compare the *in vivo* antitumor activity of CART and CPR/CART with CD28 versus 41BB costimulation, we utilized an orthotopic xenograft model of LN229-GBM. Orthotopic tumors were established by stereotactic injection of LN229.eGFP.Ffluc cells (day 0) using a previously described method (37, 39, 40). In the representative experiment shown (Fig. 7A), despite the substantial disease burden prior to treatment (*P* > 0.99, Kruskal–Wallis test; Fig. 7B), tumor response was observed in CART-treated cohorts, as determined by BLI. Tumor regression induced by the first T-cell dose (1 × 10^6^ CAR^+^ T-cell) was most marked in mice receiving CARζ/CPR41BB cells (*n* = 7; Fig. 7C), resulting in a significantly lower median disease burden relative to pretreatment assessment (*P* < 0.0001, two-way ANOVA with the Tukey test). Although CAR41BBζ (*n* = 5) showed effective antitumor activity, there was no significant difference in the median tumor volume following the first T-cell dose (*P* = 0.3; Mann–Whitney test) when compared with control treatment with CAR28ζ (*n* = 5) unlike CARζ/CPR41BB cells (*P* = 0.01, Mann–Whitney test). The tumor responses achieved were sustained in all groups for prolonged periods following a second intratumoral T-cell injection (2 × 10^6^ CAR^+^ T-cell), as shown by serial assessment of tumor burden (Fig. 7D). Untreated mice (*n* = 3) showed exponential tumor growth and either died or reached euthanasia endpoints by day +54 after tumor inoculation. The median follow-up for all surviving mice in the treatment cohorts was 124 days (range: 25–321 days). The treatment effect was measured by assessment of time-to-progression, determined by BLI after treatment (up to 150 days after the first T-cell dose). All mice treated with HER2-specific CART had survival benefit without tumor progression (Fig. 7E) compared with no treatment (*P* < 0.01, log-rank test, Holm–Sidak method) or sham treatment with NT T cells (*P* < 0.05, log-rank test, Holm–Sidak method). Tumor recurrences were the lowest in the CARζ/CPR41BB cohort (HR: vs. LN229-GBM = 0.03, 95% confidence interval, 0.0008–0.22; vs. NT = 0.075, 95% confidence interval, 0.014–0.4), with six of seven (86%) mice surviving without measurable tumor progression, compared with 20% in the CAR28ζ-treated group and up to 40% in the CAR41BBζ-treated group. The mice receiving control treatment, CAR28ζ (*n* = 5), showed improved PFS over sham treatment with NT T cells (*n* = 6; *P* = 0.01, log-rank test) and no treatment (*n* = 3; *P* = 0.008, log-rank test). Upon comparing CAR28ζ with experimental treatments, we found that mice receiving CARζ/CPR41BB cells (*n* = 7) had a PFS advantage (*P* = 0.04, log-rank test), whereas mice receiving CAR41BBζ cells (*n* = 5) did not (*P* = 0.89, log-rank test).

### CARζ/CPR41BB cells curb osteosarcoma metastasis without overt toxicity

To examine the effect of sustained signal 2 mediated by abundant PD-L1 on effector functions of systemically delivered CARζ/CPR41BB cells, we chose an experimental osteosarcoma model. *In vitro*, osteosarcoma cells exhibited PD-L1 expression dynamics (Supplementary Fig. S9A) similar to that of GBM, and these HER2^+^ tumor targets (Supplementary Fig. S9B) were lysed by CARζ/CPR41BB cells (Supplementary Fig. S9C). For *in vivo* evaluation of the CARζ/CPR41BB antitumor activity, we used an aggressive lung metastatic model of osteosarcoma (32), established by intratibial injection of 143B.eGFP.Ffluc cells in NSG mice (Supplementary Fig. S10A). The mice carrying a large disease burden, confirmed by BLI, were randomized on day +5 after tumor inoculation (Supplementary Fig. S10B) to receive a relatively small dose of CART (5 × 10^6^ T-cell) delivered by intraperitoneal injection. Under these strained experimental conditions, treatment with CARζ/CPR41BB cells (*n* = 5) resulted in improved overall survival (Supplementary Fig. S10C) compared with untreated (*n* = 5; *P* = 0.002, log-rank test) mice. To assess potential systemic adverse effects resulting from PD-L1–mediated excessive activation of CPR/CART, we treated mice with established 143B osteosarcoma xenografts using two intraperitoneal injections of 10 × 10^6^ CARζ/CPR41BB (*n* = 7) or CAR41BBζ (*n* = 5) cells on day +5 and day +13 after tumor inoculation (Supplementary Fig. S10D). We observed no differences in overall well-being or weight when examined over time (Supplementary Fig. S10E). Two weeks after the first T-cell dose, BLI demonstrated a lower metastatic burden in the lungs of mice treated with CARζ/CPR41BB cells compared with those treated with CAR41BBζ (Supplementary Fig. S10F). These findings were further confirmed by lung histopathology. We assessed the trafficking of CART (human CD3^+^ cells) to intratibial primary tumors (Supplementary Fig. S11A and S11B) and examined both the primary tumors and lung metastases for PD-L1 expression (Supplementary Fig. S11C) using IHC. Lung histopathology from mice treated with CARζ/CPR41BB cells did not show evidence of alveolar or interstitial tissue damage secondary to immune effector–mediated inflammation (Supplementary Fig. S11D). Congruent with the *in vitro* and *in vivo* characterization in GBM models, CARζ/CPR41BB cells consistently showed robust activity against osteosarcoma without evident toxicities from continued engagement of PD-L1 in the presence of widespread tumor burden.

## Discussion

We describe the functional differences arising from structural variations in CAR and CPR designs, demonstrating that the 41BB endodomain offers an advantage over CD28 in PD-1 CPR design when CPR is coexpressed with a first-generation CAR to achieve optimal T-cell activation. Through methodical assessment of CPR/CART permutations derived from our clinically tested FRP5-scFv–based HER2-CAR, we show that the CPR curtails PD-1/PD-L1–mediated T-cell inhibition and that the primary antigen–specific signal initiated by the CAR, along with a discrete costimulatory signal mediated by the CPR, enhances CART characteristics and antitumor activity. We refined the CPR/CART design to fine-tune the effector functions and evaluated their *in vivo* activity after locoregional and systemic delivery in two distinct orthotopic tumor models. The unique pattern of CARIS formation and the specific cytokine profile of CARζ/CPR41BB cells, likely resulting from balanced dual activation through CAR and CPR ligation, conferred a favorable metabolic signature and augmented their antitumor activity in animal models. Our results suggest that decoupling T-cell activation signals through CPR41BB improves T-cell effector function compared with conventional second-generation CART, in which cytoplasmic domains for both signal 1 and signal 2 are integrated into the CAR. This approach could also shield adoptively transferred CART from immune-suppressive signals common in many solid cancers.

Our studies using primary human GBM tumors demonstrate that the PD-1 pathway likely plays a role in resistance to CART. Others have shown variable expression of PD-L1 on GBM (41), and higher expression may portend worse prognosis (42). PD-L2, a second ligand for PD-1, is present on GBM cells and infiltrating immune cells and may also confer an unfavorable prognosis (55, 56). Like the effect on PD-L1, IFN-γ promotes PD-L2 expression in some tumors (68). Consequently, the abundance of PD-L1/PD-L2 in GBM and regulation of their expression by effector cytokines offer an opportunity to redirect the immune checkpoint axis to achieve optimal CART activation within the TME, conceptually mimicking coordinated T-cell activation through the TCR complex. Upon engaging PD-L1 or PD-L2, the extracellular domain of the CPR, derived from the native PD-1, allows for continued costimulatory signaling to promote CART proliferation, expansion, and sustained activation, which are otherwise lacking in solid tumors in part due to the absence of costimulatory ligands. The essential role of PD-1 in maintaining immune-tolerance decreases the probability of downregulation of its ligands; therefore, loss of CPR-mediated costimulation is less likely, providing a potential advantage over CART utilizing a tumor antigen as a costimulatory ligand. Coexpression of the CAR and CPR using a single bicistronic vector simplifies the clinical implementation and assures comparable expression of both.

Balancing antitumor function with immune effector–mediated toxicity is crucial for the effective use of CART in solid tumors, especially when targeting shared antigens like HER2, given the risk of on-target, off-tumor toxicity. In this study, we found that decoupling the primary T-cell activation and costimulatory signals decreases proinflammatory cytokine production by CART while preserving their short-term and long-term antitumor activity. Maximizing CART efficacy relies on fine-tuning signal strength to enhance T-cell proliferation and cytotoxicity but without inducing functional exhaustion. How these T-cell attributes are affected by the configuration of signal 1– and signal 2–initiating endodomains is not clearly delineated in CART. The phenotypic and mechanistic differences we observed among CPR/CART varied not only by the type of costimulatory domain incorporated but also by the mode of signal delivery. Given these variables, we performed vigorous comparative functional testing *in vitro* and *in vivo*, identifying CARζ/CPR41BB cells as the ideal candidate for clinical testing.

In our preclinical studies, CARζ/CPR41BB cells showed lower CAR-CD3ζ phosphorylation at rest compared with CAR28ζ cells and exhibited reduced cytokine production upon target encounter compared with second-generation CART containing CD28 or 41BB. However, CARζ/CPR41BB cells formed a cytotoxic immune synapse with GBM cells that was comparable with second-generation CAR41BBζ cells. A prior report has shown that PD-L2 is the preferred ligand for PD-1 recruitment to the immune synapse between T cells and dendritic cells (45). We found that the CPR was recruited to the CARIS with both the WT and PD-L1 KO GBM cells, but its intensity was dependent on the presence of PD-L1 on tumor cells. On the other hand, CPR intensity correlated with that of CAR, with propensity for increased recruitment of CPR later in the synapse formation. This suggests that CPR may simply follow the kinetics of native PD-1 accumulation at the immune synapse (45) and could have a competitive advantage due to high levels of forced expression. However, this was not experimentally demonstrated due to limitations of the methodology used in differentiating the CPR extracellular domain from native PD-1 at the CARIS. The structural and subcellular localization of molecules at the CARIS could be studied by confocal microscopy (13, 37, 69), but this is a low-throughput method and may introduce selection bias during data acquisition from nonhomogeneous primary T-cell samples. CAR costimulatory signals impact T-cell metabolism, which in turn influences the CART differentiation, antitumor function, and longevity (66, 70). In our study, although the metabolic signatures of CARζ/CPR41BB and CAR41BBζ were similar at rest and following short-term activation, we observed that CARζ/CPR41BB cells exhibited a measurable preference for mitochondrial metabolism over glycolytic pathways following prolonged antigen encounter.

T-cell functions are shown to be differentially sensitive to PD-1–mediated inhibition, with IL-2 and TNF-α production being most susceptible followed by IFN-γ production and cytotoxicity (53). In line with this finding, we observed a PD-L1 density–dependent decrease and increase in effector cytokine production by CART and CPR/CART, respectively, with the effects of counteracting the PD-1 axis most marked on IL-2 production. These results are further supported by the cytotoxic activity and cytokine secretion profile of CPR/CART upon encountering autologous GBM cells. T-cell are known to be inherently dysfunctional in patients with cancer and are particularly so in patients with GBM (28–31, 71). In this study, we demonstrated the potential for CPR to augment the antitumor activity of patient-derived CART against matched autologous GBM cells, thereby lending strength to our findings and supporting their clinical applicability. The tumor-associated antigen HER2 is overexpressed in several other solid cancers (23, 34, 72–81), expanding the prospects for therapeutic application of CARζ/CPR41BB cells. Thus, we substantiated our key findings in an independent systemic model of HER2^+^ human cancer by studying the dynamic surface expression of PD-L1 in osteosarcoma and the antitumor function of CARζ/CPR41BB cells *in vitro* and after adoptive transfer in a 143B osteosarcoma model. Additionally, in this model, we confirmed homing of CARζ/CPR41BB cells to HER2^+^/PD-L1^+^ tumors, resulting in lower metastatic burden in the lungs without immune effector–mediated tissue damage evident on lung histopathology. Although our T cells–intrinsic approach to checkpoint modulation shows promise in overcoming some limitations of CART for solid tumors, such as immune inhibition and functional exhaustion, the full impact of the CPR is difficult to study comprehensively *in vivo* in the preclinical setting due to lack of appropriate models. For instance, compared with adjunct immune checkpoint inhibitor therapy in combination with CART, the endogenous T-cell response may be limited when using a CPR and may not effectively elicit epitope spreading needed to overcome tumor heterogeneity. Whereas syngeneic models using human HER2-expressing transgenic mouse glioma cells may allow for evaluating the effects of CPR on endogenous immune cells in the TME, human protein expression could alter tumor immune infiltrates, and the need to use mouse T cells makes direct extrapolation of findings for translational applications challenging. Our study did not evaluate other mediators of the TME, which may circumvent or counteract the positive effects of the CPR on CART. Comparative analysis of additional CPR/CART designs, including CAR41BBζ with CPR coexpression, could have provided further insights into the mechanistic differences. Although the inclusion of extended designs with additional combinations of transmembrane and signaling domains, as well as control conditions, was limited by the availability of patient materials in early experimental studies, the results did not suggest an advantage for the CAR41BBζ platform over our clinically tested CAR28ζ. Given these limitations, the mechanistic attributes should be evaluated in CART targeting other tumor antigens using additional CPR/CART designs to assess their broader applicability. Nevertheless, the potential advantages demonstrated by our studies support the continued clinical development and testing of HER2-targeting CARζ/CPR41BB T-cell in a phase 1 clinical trial.

## Supplementary Material

Supplementary Figure 1 PD-L1 expression in glioblastoma (GBM) cells and PD-1 upregulation in CART.

Supplementary Figure 2 Analysis of CAR immune synapse (CARIS) with HER2+ GBM cells.

Supplementary Figure 3 Expression of CAR/CPR on T cells and HER2/PD-L1 on tumor cells.

Supplementary Figure 4 Summary of CPR/CART design and characterization of CARζ co-expressing CPR containing CD28 or 4-1BB signaling.

Supplementary Figure 5 Immunophenotype and immune-checkpoint receptor expression in patient-derived CPR/CART cells.

Supplementary Figure 6 Immunophenotype and functional profile of CARζ/CPR41BB in comparison to CAR41BBζ cells.

Supplementary Figure 7 Analysis of CAR immune synapse (CARIS) formation by CARζ/CPR41BB and CAR41BBζ cells with wild-type and PD-L1 KO LN229-GBM cells.

Supplementary Figure 8 Assessment of mitochondrial metabolism in CARζ/CPR41BB cells.

Supplementary Figure 9 Assessment of PD-L1 expression and CARζ/CPR41BB cell cytotoxic function in osteosarcoma.

Supplementary Figure 10 In vivo function of systemically administered CARζ/CPR41BB cells in a metastatic osteosarcoma model.

Supplementary Figure 11 Immunohistochemistry and histopathology findings in a metastatic osteosarcoma model after treatment with CARζ/CPR41BB cells.

Supplementary Table 1 Summary of CAR and CPR constructs evaluated in functional assays
