# Supplementary material for: A Checkpoint Reversal Receptor Mediates Bipartite Activation and Enhances CAR T-cell Function
Source: Cancer Res Commun. 2025 Mar 31;5(3):527–48. doi: 10.1158/2767-9764.CRC-24-0125 (PMC11955954; doi:10.1158/2767-9764.CRC-24-0125)
Supplement: Supplementary Figure 1 — PD-L1 expression in glioblastoma (GBM) cells and PD-1 upregulation in CART. [file crc-24-0125_supplementary_figure_1_suppsf1.pdf]

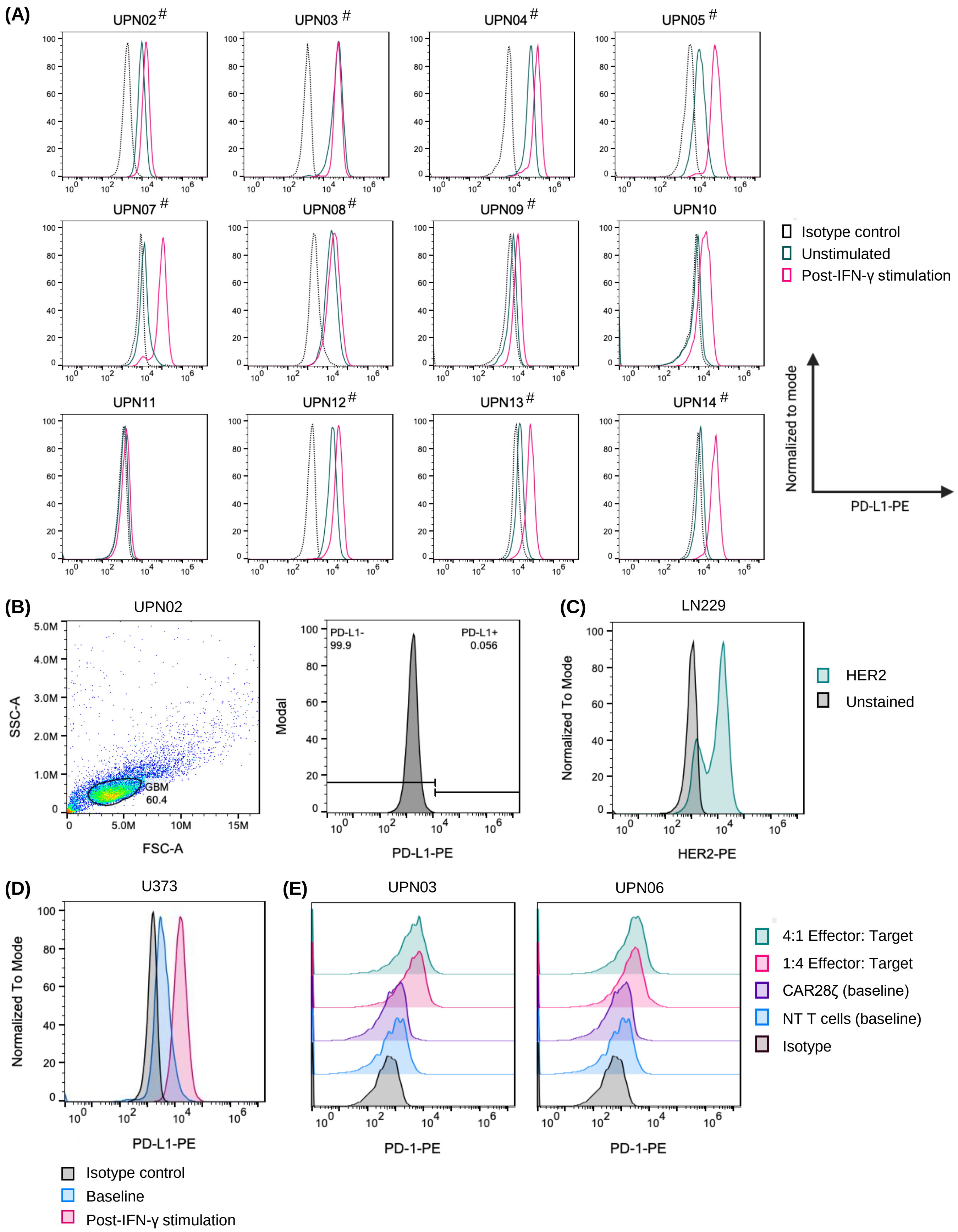

Supplementary Figure 1

Supplementary Figure 1: PD-L1 expression in glioblastoma (GBM) cells and PD-1 upregulation in CART. (A) Surface expression of PD-L1 in primary GBM samples at baseline and 24-48 hours after IFN-γ (10 ng/mL) exposure. UPN, Unique Patient Number. #represents tumor samples with constitutive PD-L1 expression. **(B)** Gating strategy for PD-L1 expression on primary GBM samples. Representative isotype control sample from UPN02 with PD-L1 gate shown. **(C)** Histogram demonstrating HER2 expression on LN229-GBM cells detected by flow cytometry. **(D)** PD-L1 expression in U373-GBM cells at baseline and after 48 hours of stimulation with exogenous IFN-γ. **(E)** PD-1 surface expression on T cells at baseline (*purple*) and after 48 hours of co-culture with primary $HER2^+$ GBM cells (from UPN03 and UPN06) at different effector to target ratios (*pink and green*). The baseline controls shown in both panels, including the isotype (*grey*) and non-transduced (NT) T cells (*blue*), are from the same T-cell donor.
