# Supplementary material for: A Checkpoint Reversal Receptor Mediates Bipartite Activation and Enhances CAR T-cell Function
Source: Cancer Res Commun. 2025 Mar 31;5(3):527–48. doi: 10.1158/2767-9764.CRC-24-0125 (PMC11955954; doi:10.1158/2767-9764.CRC-24-0125)
Supplement: Supplementary Figure 2 — Analysis of CAR immune synapse (CARIS) with HER2+ GBM cells. [file crc-24-0125_supplementary_figure_2_suppsf2.pdf]

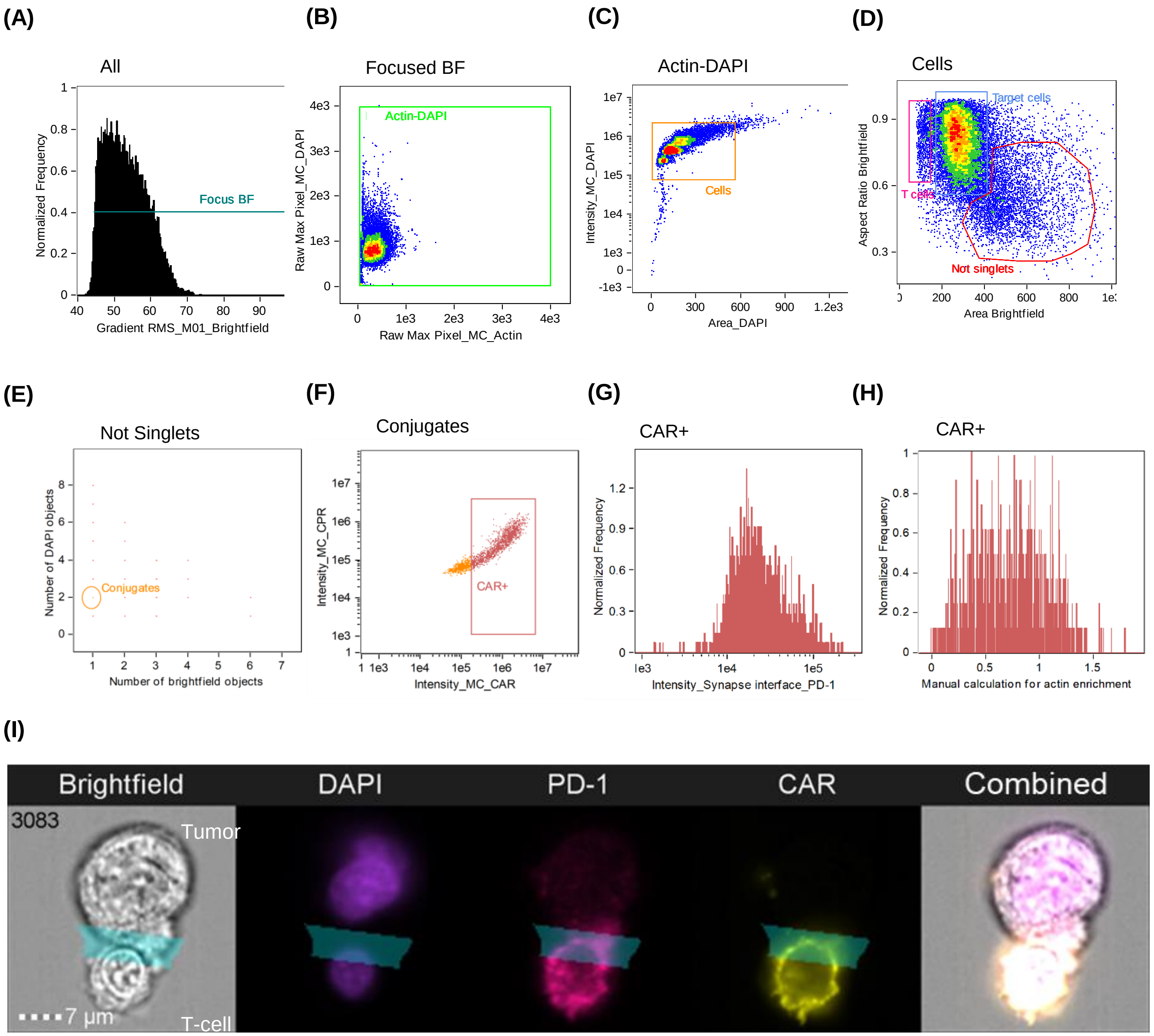


**Supplementary Figure 2**

**Supplementary Figure 2: Analysis of CAR immune synapse (CARIS) with HER2$^{+}$ GBM cells. (A)** to **(I)** show a representative example of event capture and gating strategy for CAR$^{+}$ T-cell and GBM conjugates on Amnis ImageStream MKII taken from a co-culture of wild-type (WT) LN229-GBM and CART at 30 minutes. **(A)** In focus events based on root mean square (RMS>45) selected and **(B)** ≥20,000 in-focus actin and DAPI double-positive cells were collected for every sample. **(C)** Cells further gated from large aggregates, cellular debris, and non-cellular events using the area versus the mean intensity of the DAPI signal per cell. **(D)** Cell conjugates identified based on their area to aspect ratio in the brightfield channel (alongside with irrelevant populations of singular T cells and target cells. **(E)** Healthy conjugates selected by DAPI object count where events have two separate nuclei labeled but appear as one item in brightfield. This allows the segregation between true conjugates (i.e., 2 identifiable nuclei within 1 single brightfield object due to the presence of the immune synapse bridging the 2 cells) versus two (or more) cells captured in the same image but not interacting with each other. **(F)** Conjugates with CAR$^{+}$ T cells selected based on single-stain controls. **(G)** An unbiased mask called 'Valley' based on the dip of intensity between the nuclei (DAPI channel) of the T-cell and the target cell was used to identify immune synapse area. Intensity of PD-1 measured within the immune synapse area. **(H)** The same masked area is further used for actin intensity measurement. **(I)** Representative images of conjugate and unbiased immune synapse mask placement (area in light cyan). The area highlighted in cyan on top of the actin and CAR channels indicates the area defined as the CAR immune synapse (CARIS) where the measurement of PD-1 and CPR intensity was performed.
