# Supplementary material for: A Checkpoint Reversal Receptor Mediates Bipartite Activation and Enhances CAR T-cell Function
Source: Cancer Res Commun. 2025 Mar 31;5(3):527–48. doi: 10.1158/2767-9764.CRC-24-0125 (PMC11955954; doi:10.1158/2767-9764.CRC-24-0125)
Supplement: Supplementary Figure 3 — Expression of CAR/CPR on T cells and HER2/PD-L1 on tumor cells. [file crc-24-0125_supplementary_figure_3_suppsf3.pdf]

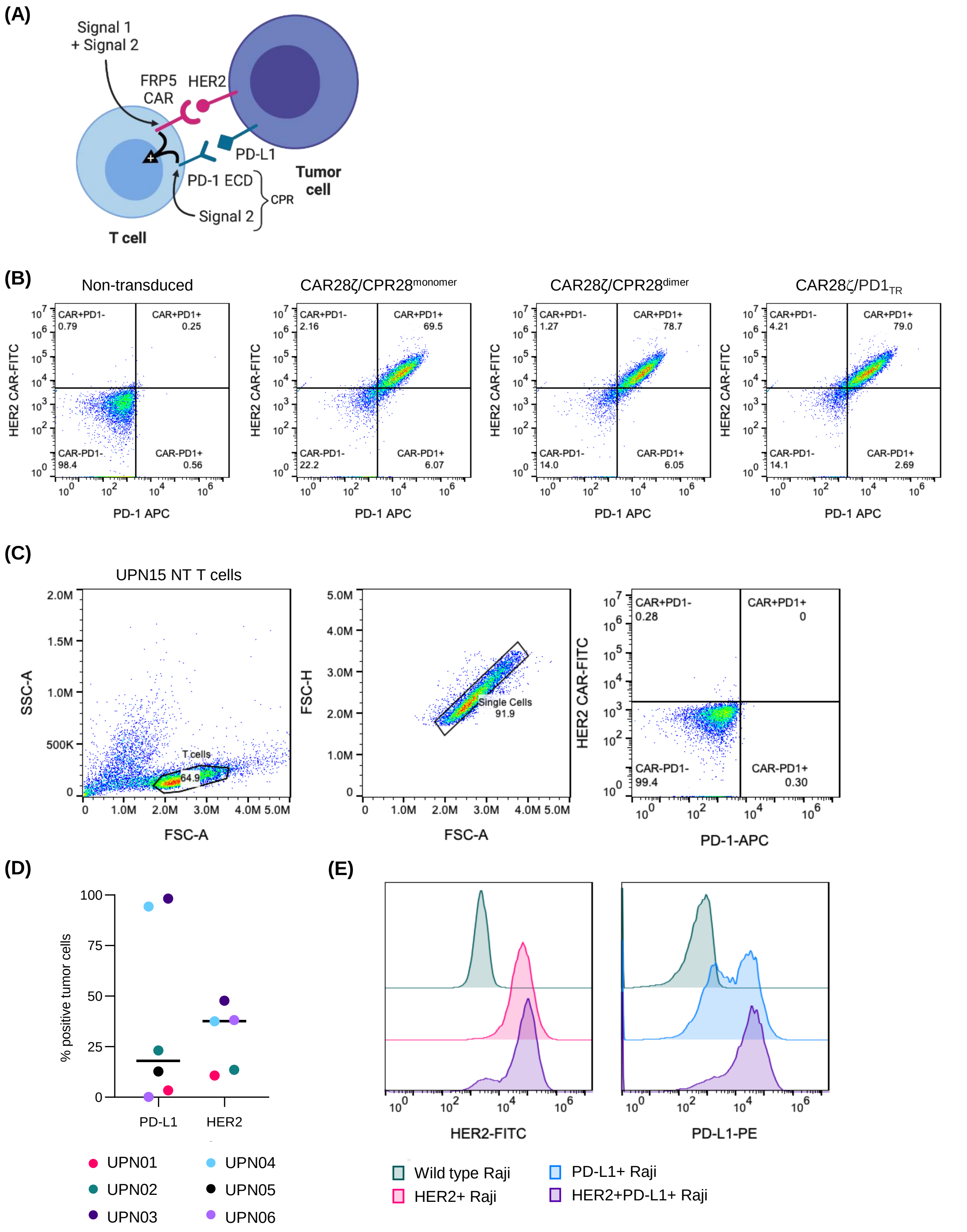

Supplementary Figure 3

Supplementary Figure 3: Expression of CAR/CPR on T cells and HER2/PD-L1 on tumor cells. (A) Illustration of the signal delivery for T-cell activation upon CAR28ζ and CPR28 engagement with respective targets expressed on a tumor cell. **(B)** Detection of HER2-CAR and PD-1 CPR expression (using anti-PD-1 antibody) on CAR28ζ/CPR28^{monomer}, CAR28ζ/CPR28^{dimer}, and CAR28ζ/PD1$_{TR}$ T cells by flow cytometry. Non-transduced T cells were used as controls. Representative panel from one donor shown. **(C)** Gating strategy to determine HER2-CAR and PD-1 expression using flow cytometry. Representative sample shown from UPN15. **(D)** The expression of HER2 antigen and PD-L1 on primary GBM cells used in functional evaluations of CPR/CART. Data shown as individual values. The horizontal line represents the median. **(E)** HER2 (*left panel*) and PD-L1 (*right panel*) expression on Raji cells transduced with HER2 or PD-L1 or both proteins and sorted using flow cytometry.
