# Supplementary material for: A Checkpoint Reversal Receptor Mediates Bipartite Activation and Enhances CAR T-cell Function
Source: Cancer Res Commun. 2025 Mar 31;5(3):527–48. doi: 10.1158/2767-9764.CRC-24-0125 (PMC11955954; doi:10.1158/2767-9764.CRC-24-0125)
Supplement: Supplementary Figure 4 — Summary of CPR/CART design and characterization of CARζ co-expressing CPR containing CD28 or 4-1BB signaling. [file crc-24-0125_supplementary_figure_4_suppsf4.pdf]

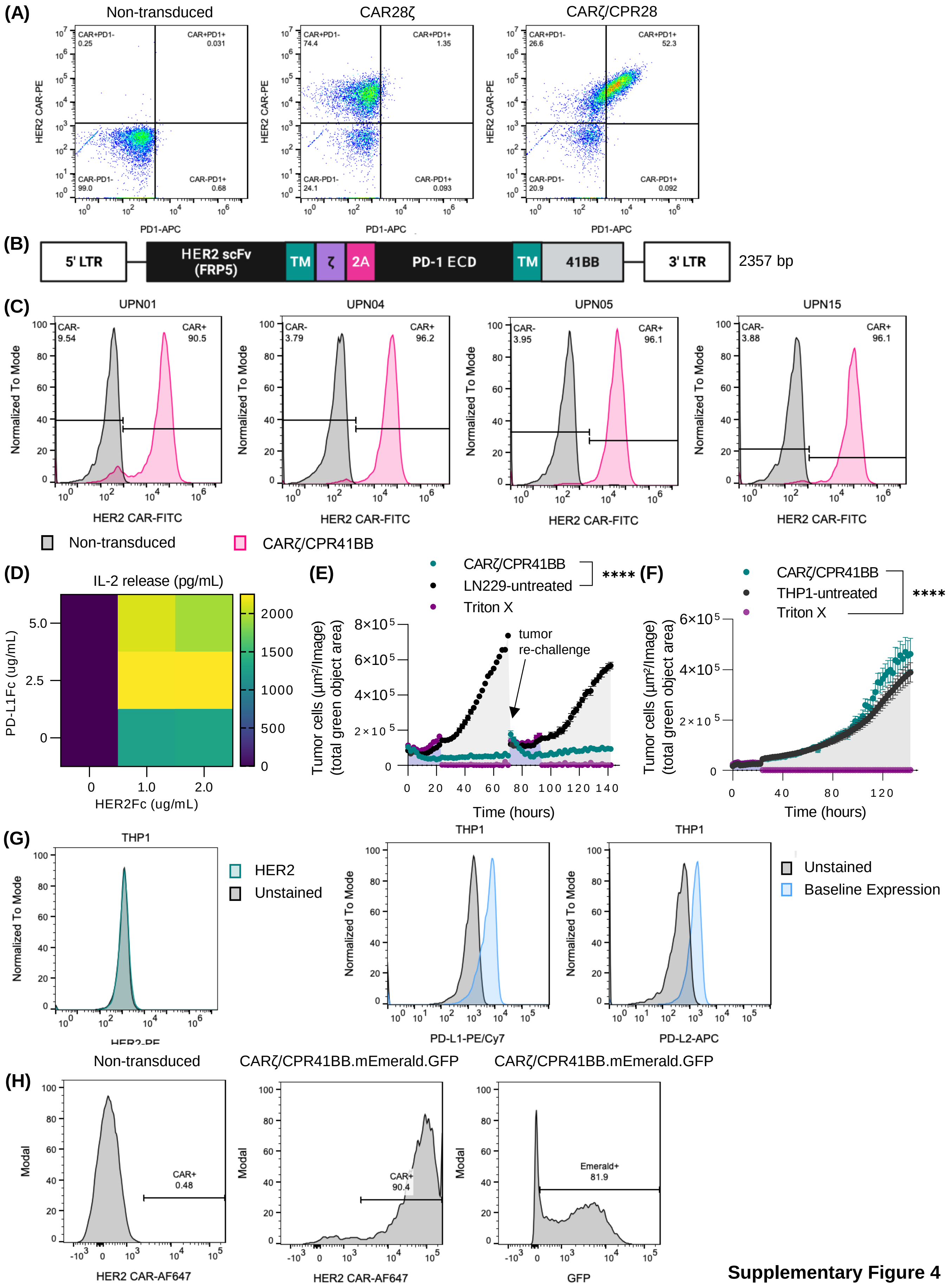

Supplementary Figure 4

Supplementary Figure 4: Summary of CPR/CART design and characterization of CARζ co-expressing CPR containing CD28 or 4-1BB signaling. (A) Dot plots demonstrating HER2-CAR and PD-1 expression on non-transduced (*left panel*), CAR28ζ (*middle panel*), and CARζ/CPR28 (*right panel*) cells assessed by flow cytometry. Representative results from one donor shown. The shift to the right in PD-1 expression represents CPR expression in CARζ/CPR28 cells. **(B)** Schematic representation of the bicistronic vector encoding for CARζ/CPR41BB. **(C)** HER2-CAR expression on patient-derived CARζ/CPR41BB cells. **(D)** CARζ/CPR41BB cells show HER2-specific activation, with preserved IL-2 production at higher PD-L1Fc concentrations. **(E)** CARζ/CPR41BB cells co-cultured with GFP-labeled LN229-GBM cells (T-cell to tumor cell ratio of 1:5) for 72 hours demonstrate robust antitumor activity when re-challenged with fresh tumor cells at the same ratio in an Incucyte® live-cell assay. At hour 72, both the test and control tumor-only conditions were replaced with fresh tumor cells. Triton X was used as a positive control for target cell lysis. **(F)** CARζ/CPR41BB cells did not lyse human monocytic leukemia cell line THP1, which are **(G)** $HER2^-$ (*left panel*), $PD\text{-}L1^+/PD\text{-}L2^+$ (*right panel*). **(H)** Representative histograms from flow cytometry analysis demonstrating proportional expression of HER2-CAR and mEmerald-GFP-tagged CPR molecules on CARζ/CPR41BB.mEmerald.GFP cells.
