# Supplementary material for: A Checkpoint Reversal Receptor Mediates Bipartite Activation and Enhances CAR T-cell Function
Source: Cancer Res Commun. 2025 Mar 31;5(3):527–48. doi: 10.1158/2767-9764.CRC-24-0125 (PMC11955954; doi:10.1158/2767-9764.CRC-24-0125)
Supplement: Supplementary Figure 5 — Immunophenotype and immune-checkpoint receptor expression in patient-derived CPR/CART cells. [file crc-24-0125_supplementary_figure_5_suppsf5.pdf]

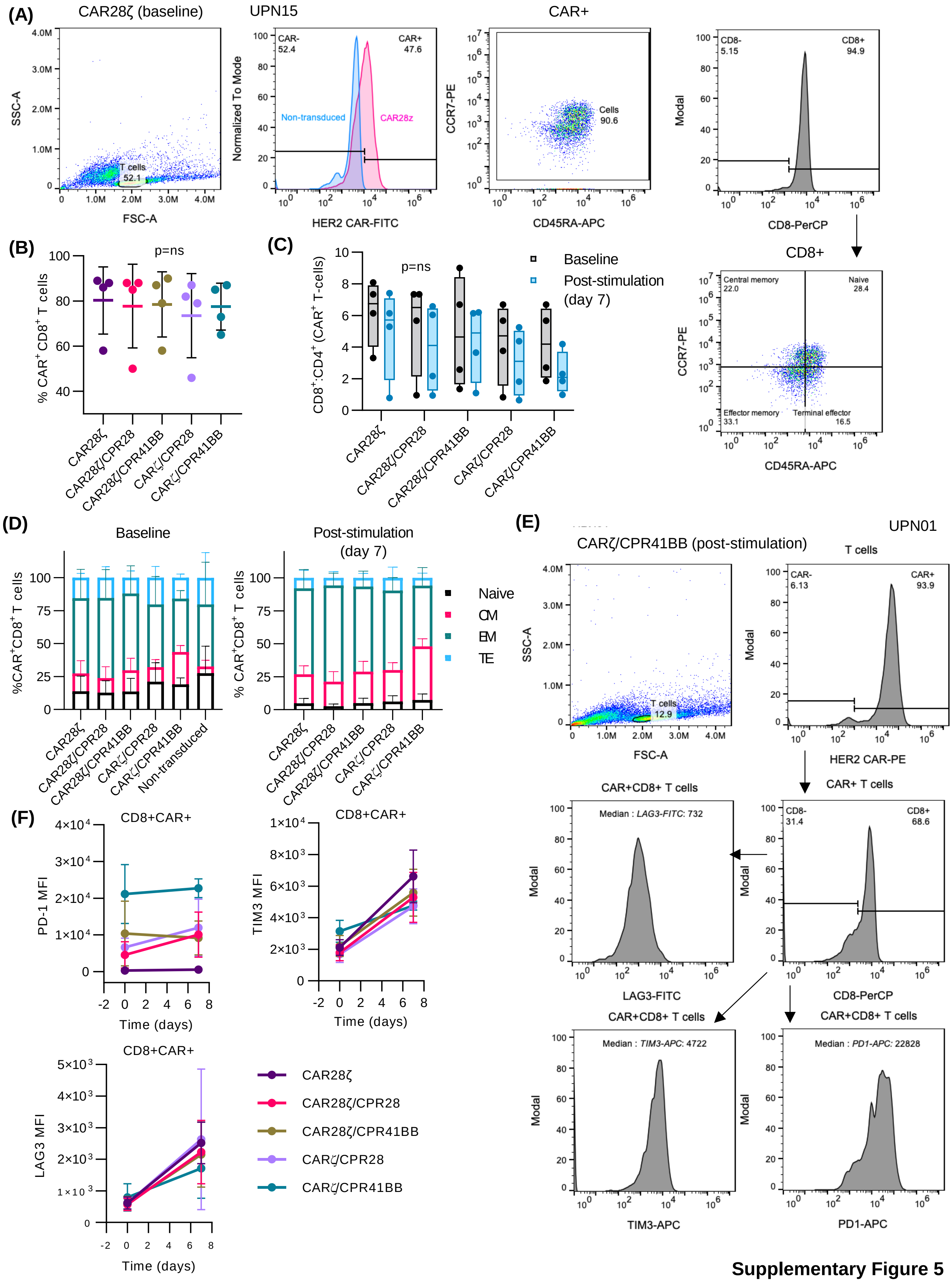


**Supplementary Figure 5**

**Supplementary Figure 5: Immunophenotype and immune-checkpoint receptor expression in patient-derived CPR/CART cells. (A)** Gating strategy for assessment of surface immunophenotype of T cells. Representative flow cytometry analysis from a patient-derived CAR28ζ product shown. **(B)** Percent (%) of $CAR^+CD8^+$ T cells in CART products generated from patients with GBM (n=4) and expanded in IL-7/IL-15. ns, $p>0.05$, One-way ANOVA with Tukey's multiple comparisons. **(C)** $CD8^+$ to $CD4^+$ ratio in CAR-expressing T cells, at baseline and at 7 days of repeated stimulation (every 48 hours) with autologous tumor cells. ns, $p>0.05$, Two-tailed student's t test. **(D)** Immunophenotype of $CAR^+CD8^+$ T cells (n=4 patients) at baseline and at 7 days of repeated stimulation with autologous GBM cells determined by flow cytometry. CM, central memory, EM, effector memory, TE, terminal effector. **(E)** Gating strategy for evaluation of immune-checkpoint receptor expression on T cells from patients with GBM following co-culture with autologous tumor cells. Flow cytometry analysis from a representative post-stimulation patient sample shown. A tight gate was placed on the T-cell population to exclude tumor cells in co-cultures (*upper left panel*). $CAR^+$ (*middle panel*), $CD8^+$ cells were selected (*right panel*), and expression of immune-checkpoint receptors was then assessed (*lower panels*). Change in expression of immune-checkpoint receptors was determined by calculating fold-change in median fluorescent intensity (MFI) before and after stimulation. **(F)** Median fluorescent intensity (MFI) of PD-1, TIM3, and LAG3 $CD8^+CAR^+$ T cells, at baseline (Day 0) and at 7 days of repeat co-culture with autologous GBM cells (n=4 patients). Data shown as Mean±SD.
