# Supplementary material for: A Checkpoint Reversal Receptor Mediates Bipartite Activation and Enhances CAR T-cell Function
Source: Cancer Res Commun. 2025 Mar 31;5(3):527–48. doi: 10.1158/2767-9764.CRC-24-0125 (PMC11955954; doi:10.1158/2767-9764.CRC-24-0125)
Supplement: Supplementary Figure 6 — Immunophenotype and functional profile of CARζ/CPR41BB in comparison to CAR41BBζ cells. [file crc-24-0125_supplementary_figure_6_suppsf6.pdf]

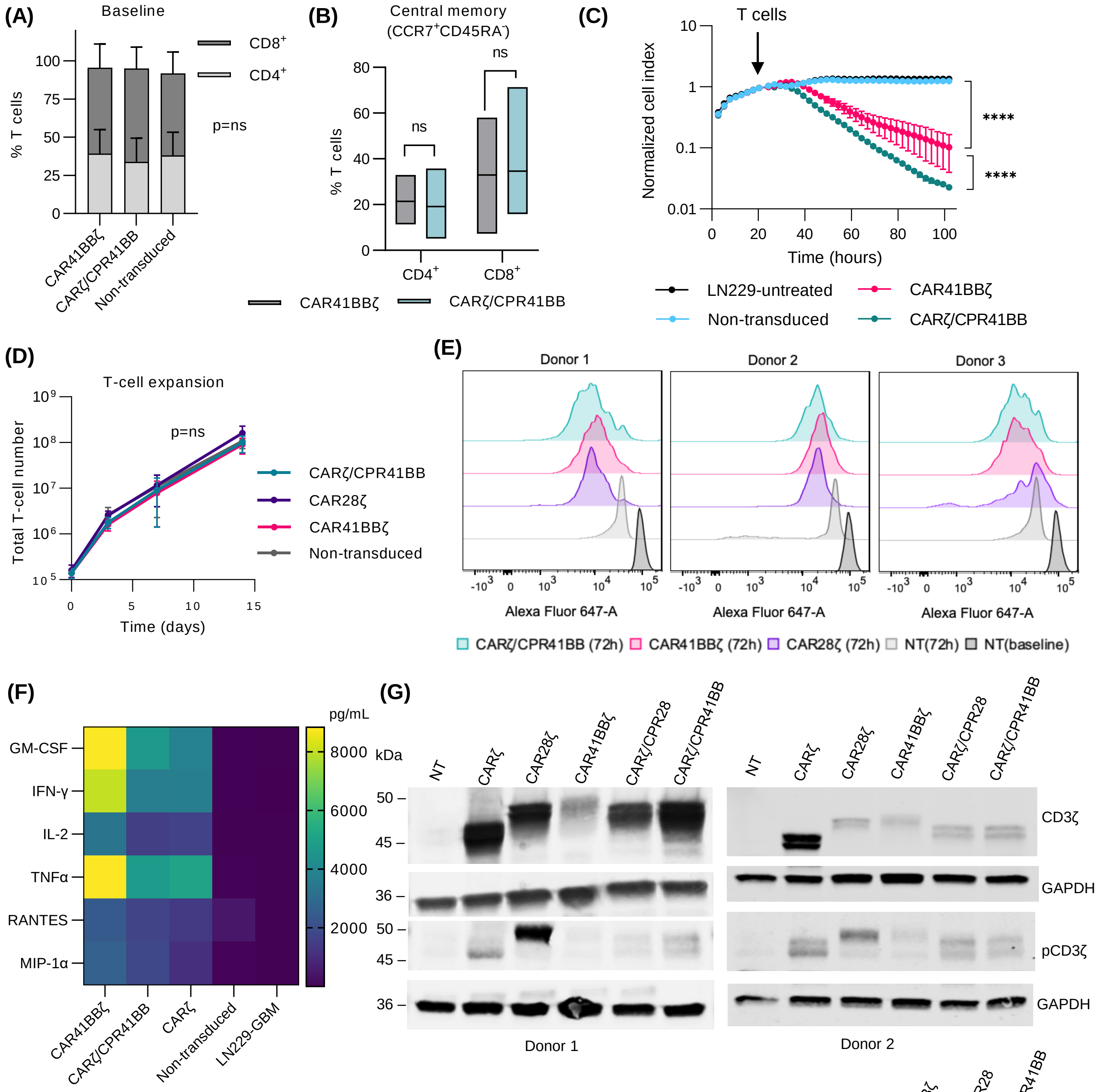

CAR T-cell	MW of CD3ζ
CARζ (FRP5.CD3ζ)	46.7 kDa
CAR28ζ (FRP5.CD28ζ)	51 kDa
CAR41BBζ (FRP5.41BBζ)	52 kDa

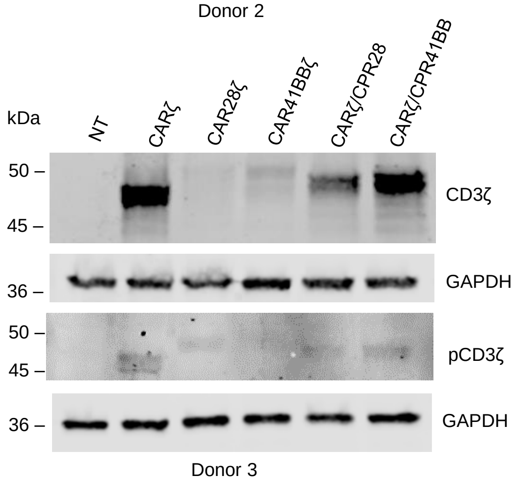

Supplementary Figure 6

Supplementary Figure 6: Immunophenotype and functional profile of CARζ/CPR41BB in comparison to CAR41BBζ cells. (A) Percent (%) of $CD4^+$ and $CD8^+$ cells at baseline (maintained in IL-7/IL-15) in CARζ/CPR41BB, CAR41BBζ, and non-transduced T cells (n=3 donors). Data shown as Mean±SD. ns, p>0.05, One-way ANOVA with Tukey's multiple comparisons. **(B)** % of $CD4^+$ and $CD8^+$ CARζ/CPR41BB and CAR41BBζ cells exhibiting a central memory ($CCR7^+CD45RA^-$) immunophenotype. ns, p>0.05, Two-tailed student's t test. **(C)** Sustained lysis of wild-type LN229-GBM by CARζ/CPR41BB (n=3 donors; T-cell:tumor =1:5) and CAR41BBζ cells in a cell-impedance based (xCELLigence) assay. Representative experiment from one donor shown. **(D)** Cell count data demonstrating T-cell (n=3 donors) expansion over 14 days of culture in media with IL-7 and IL-15. ns, p>0.05, Two-way ANOVA with Tukey's multiple comparisons. **(E)** Proliferation of CARζ/CPR41BB cells at 72 hours of co-culture with LN229-GBM cells at effector to target ratio of 1:2, compared to CAR41BBζ, CAR28ζ, and non-transduced T-cells. **(F)** Multiplex analysis of co-culture (T-cell:tumor cell=1:1) supernatants at 24 hours comparing the pattern of pro-inflammatory cytokine production by CARζ/CPR41BB to CARζ and CAR41BBζ cells. Heatmap represents the average values from two T-cell donors evaluated simultaneously. **(G)** Western blot analysis for CAR-phosphoCD3ζ (pCD3) in T cells in a resting state (maintained in culture with IL-7/IL-15). Representative gel images from three donors are shown. Table shows the molecular weight (MW) of CD3ζ in each CART evaluated.
