# Supplementary material for: A Checkpoint Reversal Receptor Mediates Bipartite Activation and Enhances CAR T-cell Function
Source: Cancer Res Commun. 2025 Mar 31;5(3):527–48. doi: 10.1158/2767-9764.CRC-24-0125 (PMC11955954; doi:10.1158/2767-9764.CRC-24-0125)
Supplement: Supplementary Figure 7 — Analysis of CAR immune synapse (CARIS) formation by CARζ/CPR41BB and CAR41BBζ cells with wild-type and PD-L1 KO LN229-GBM cells. [file crc-24-0125_supplementary_figure_7_suppsf7.pdf]

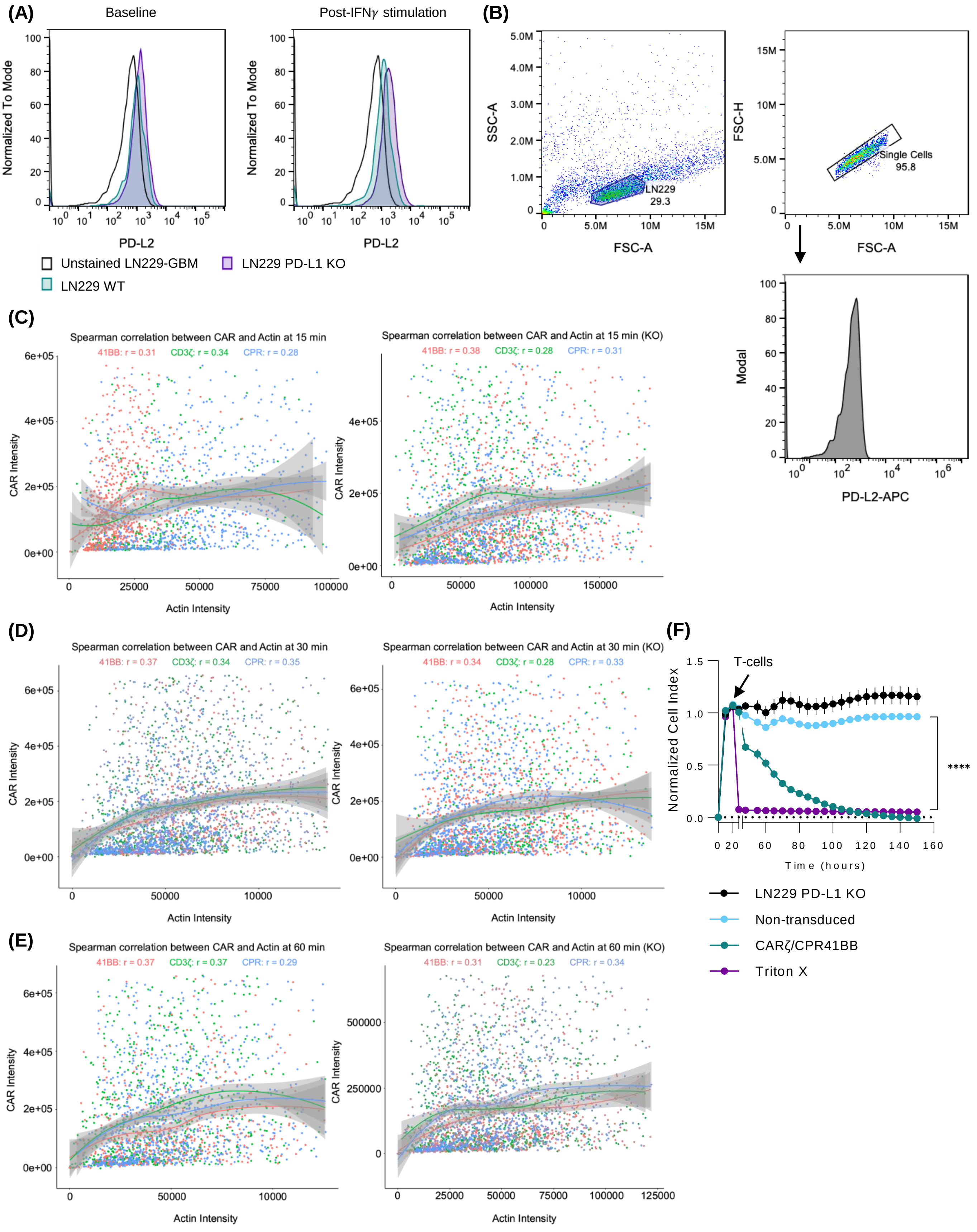

Supplementary Figure 7

Supplementary Figure 7: Analysis of CAR immune synapse (CARIS) formation by CARζ/CPR41BB and CAR41BBζ cells with wild-type and PD-L1 KO LN229-GBM cells. (A) PD-L2 expression on LN229-WT and LN229-KO cells at baseline and after 24-hour IFN-γ (10 ng/mL) exposure. **(B)** Gating strategy for PD-L2 expression on LN229-GBM cells. Representative unstained wild-type LN229-GBM sample at baseline shown. Spearman correlation between CAR intensity and actin intensity at the CARIS measured using imaging flow cytometry at **(C)** 15 minutes, **(D)** 30 minutes, and **(E)** 60 minutes of conjugation with LN229-GBM cells. 41BB represents CAR41BBζ cells, CD3ζ represents CARζ cells, and CPR represents CARζ/CPR41BB cells. **(F)** Sustained lysis of LN229-GBM PD-L1 KO by CARζ/CPR41BB (T-cell:tumor=1:5) in a cell-impedance based (xCELLigence) assay. Representative experiment from a donor shown. $^{****}p<0.0001$, Two-way ANOVA with Tukey's multiple comparisons.
