# Supplementary material for: A Checkpoint Reversal Receptor Mediates Bipartite Activation and Enhances CAR T-cell Function
Source: Cancer Res Commun. 2025 Mar 31;5(3):527–48. doi: 10.1158/2767-9764.CRC-24-0125 (PMC11955954; doi:10.1158/2767-9764.CRC-24-0125)
Supplement: Supplementary Figure 8 — Assessment of mitochondrial metabolism in CARζ/CPR41BB cells. [file crc-24-0125_supplementary_figure_8_suppsf8.pdf]

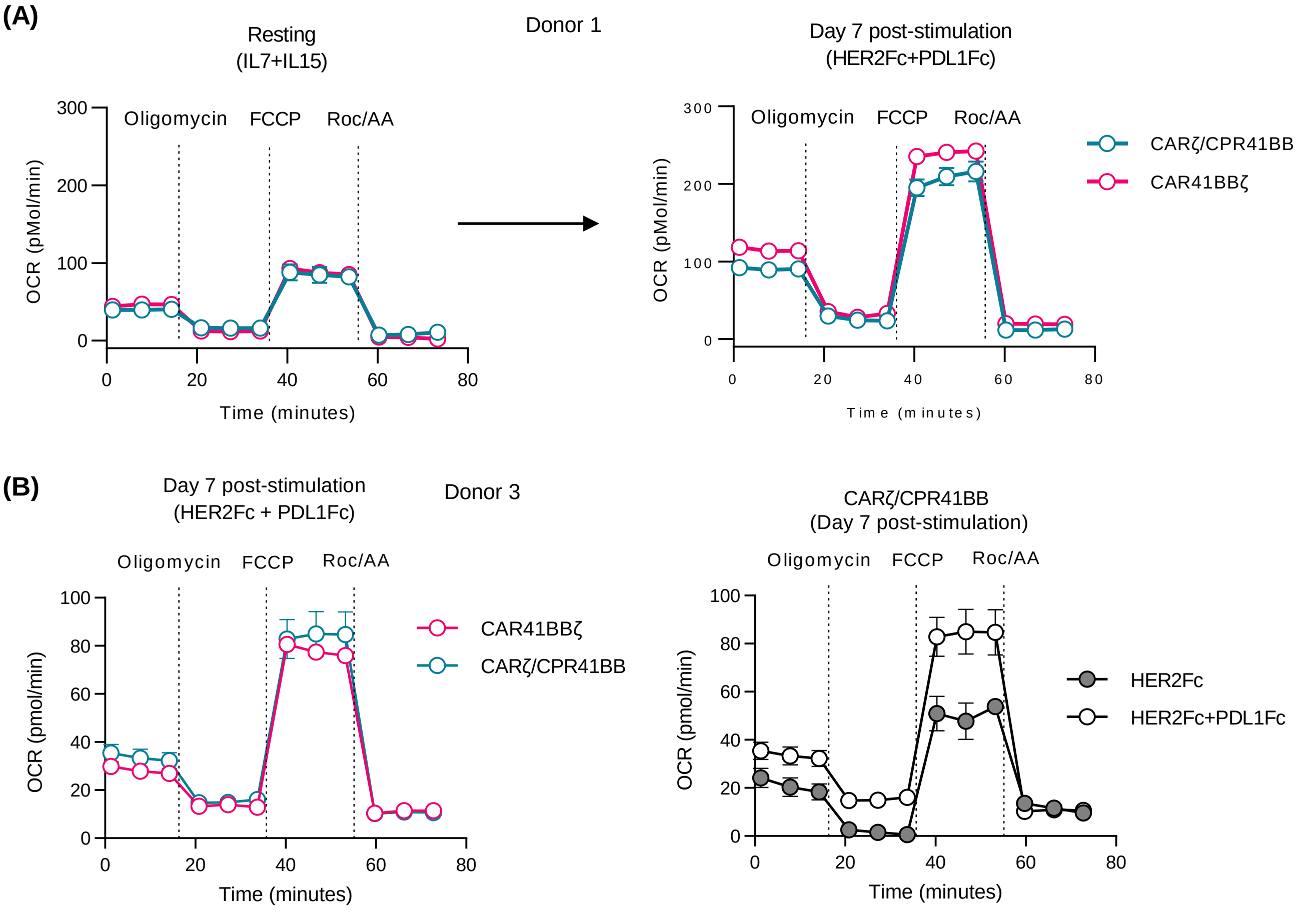

Supplementary Figure 8: Assessment of mitochondrial metabolism in CARζ/CPR41BB cells. (A) Oxygen consumption rate (OCR) of resting (*left*, in IL-7/IL-15) CARζ/CPR41BB and CAR41BBζ cells and after 7 days of continued stimulation with plate-bound HER2Fc (1 µg/mL) and PD-L1Fc (5 µg/mL) proteins. OCR was assessed under basal metabolic conditions and after sequential additions of mitochondrial inhibitors as described in Methods. Representative results from Donor 1 shown. **(B)** OCR of CARζ/CPR41BB cells stimulated with HER2Fc and PD-L1Fc proteins over days compared to CAR41BBζ cells (*left panel*) and compared to HER2Fc (1 µg/mL) stimulation alone (*right panel*). Representative results from Donor 3 shown.
