# Supplementary material for: A Checkpoint Reversal Receptor Mediates Bipartite Activation and Enhances CAR T-cell Function
Source: Cancer Res Commun. 2025 Mar 31;5(3):527–48. doi: 10.1158/2767-9764.CRC-24-0125 (PMC11955954; doi:10.1158/2767-9764.CRC-24-0125)
Supplement: Supplementary Figure 9 — Assessment of PD-L1 expression and CARζ/CPR41BB cell cytotoxic function in osteosarcoma. [file crc-24-0125_supplementary_figure_9_suppsf9.pdf]

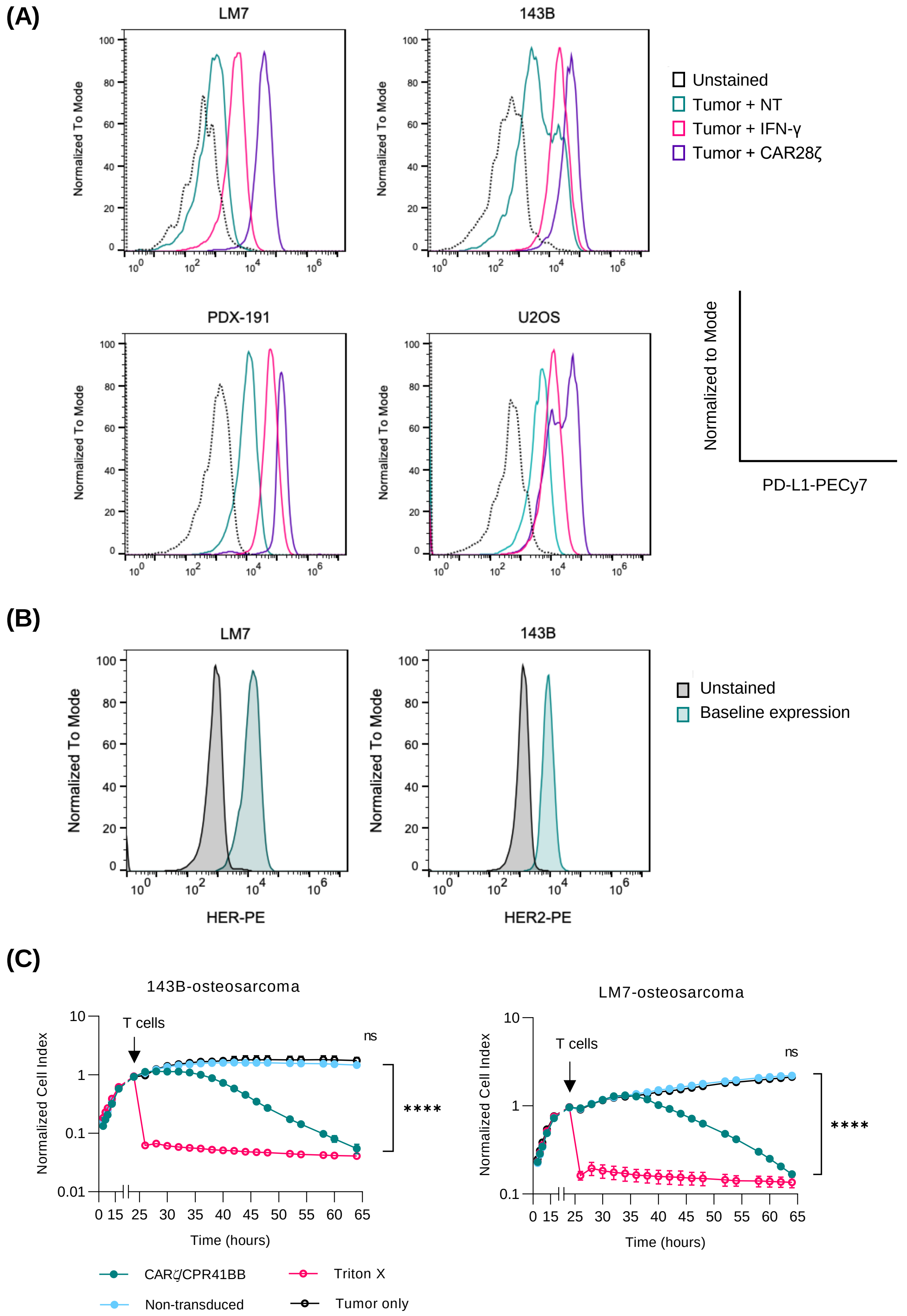

Supplementary Figure 9

Supplementary Figure 9: Assessment of PD-L1 expression and CARζ/CPR41BB cell cytotoxic function in osteosarcoma. (A) Histograms showing PD-L1 expression on sarcoma cell lines and patient-derived cells at baseline and after 72 hours of stimulation by CAR28ζ cells or IFN-γ (20 ng/mL). **(B)** Confirmation of HER2 expression in LM7- and 143B-osteosarcoma cell lines. **(C)** Assessment of long-term cytolytic ability of CARζ/CPR41BB cells against $HER2^+$ sarcoma cell lines 143B (*left panel*) and LM7 (*right panel*) using xCELLigence assay. Triton X used as positive control, and non-transduced T cells and tumor only conditions were used as negative controls. $^{****}p<0.0001$, Two-way ANOVA with Tukey's multiple comparisons.
