# Supplementary material for: A Checkpoint Reversal Receptor Mediates Bipartite Activation and Enhances CAR T-cell Function
Source: Cancer Res Commun. 2025 Mar 31;5(3):527–48. doi: 10.1158/2767-9764.CRC-24-0125 (PMC11955954; doi:10.1158/2767-9764.CRC-24-0125)
Supplement: Supplementary Figure 10 — In vivo function of systemically administered CARζ/CPR41BB cells in a metastatic osteosarcoma model. [file crc-24-0125_supplementary_figure_10_suppsf10.pdf]

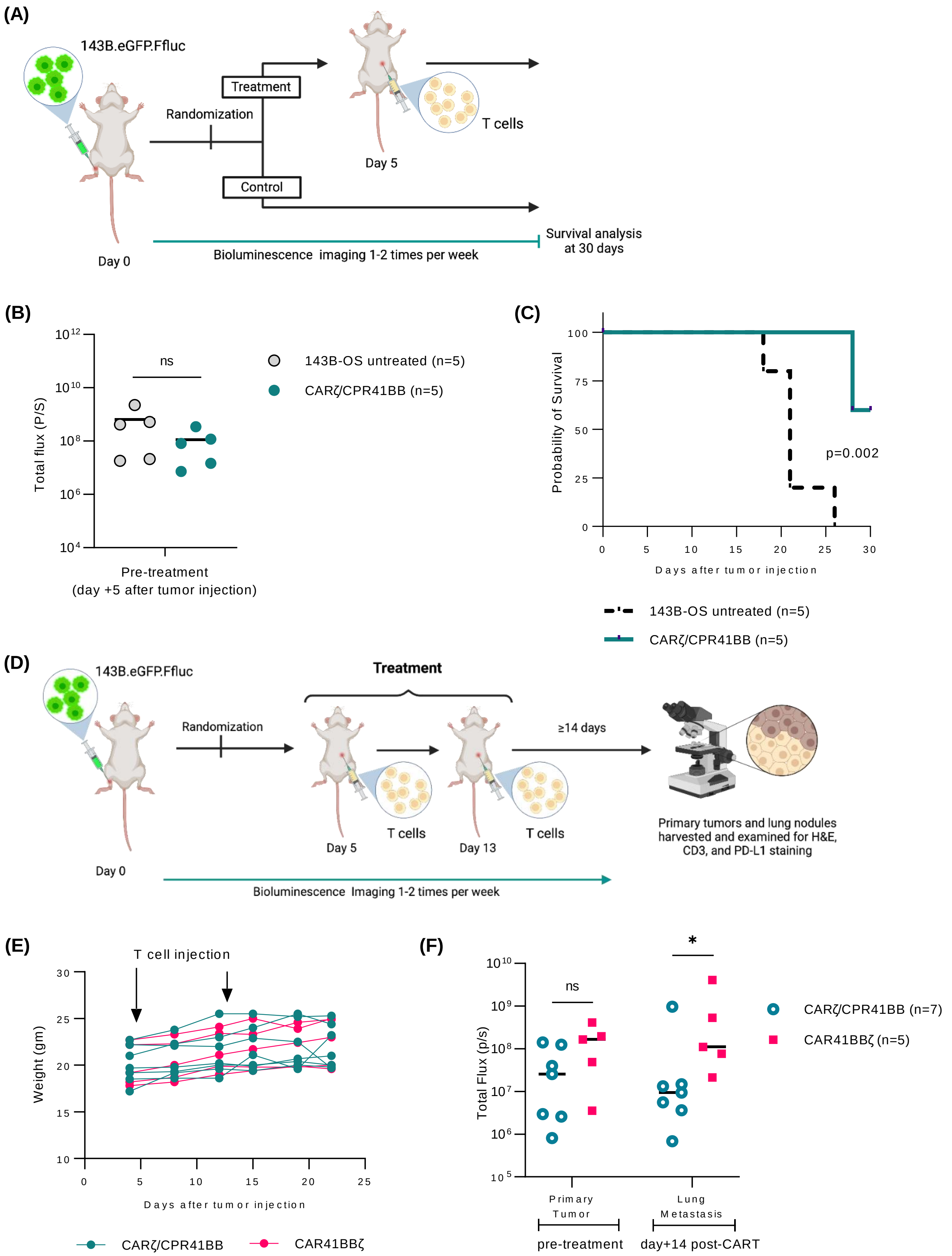

Supplementary Figure 10

Supplementary Figure 10: *In vivo* function of systemically administered CARζ/CPR41BB cells in a metastatic osteosarcoma model. (A) Experimental schema for evaluation of *in vivo* antitumor activity of CARζ/CPR41BB cells against 143B-osteosarcoma. **(B)** Pre-treatment tumor burden at the primary site measured by BLI prior to treatment in mice injected (intratibial) with eGFP.FFluc-labelled 143B-osteosarcoma cells. Individual values are shown. The horizontal line represents median. ns, not significant; Mann-Whitney test for comparison between two groups. **(C)** Kaplan-Meier overall survival analysis of mice bearing 143B-osteosarcoma xenografts following treatment with CARζ/CPR41BB (n=5) cells compared to no treatment (n=5). **p=0.002; Log-Rank (Holm-Sidak method) test for survival estimate. **(D)** Experimental schema for assessment of CARζ/CPR41BB immune effector functions in a lung metastatic 143B-osteosarcoma model after intraperitoneal injection. **(E)** Longitudinal assessment of body weight of mice bearing 143B-osteosarcoma xenografts following treatment with CARζ/CPR41BB (n=7; *green*) or CAR41BBζ (n=5; *pink*) cells. **(F)** Total flux as measured by bioluminescence imaging (BLI) for assessment of tumor burden at primary tumor site (“Pre-treatment” baseline, *left*) and pulmonary metastasis (“Day +14 post-CART”), before and after treatment with CARζ/CPR41BB (*green*) or CAR41BBζ (*pink*) cells. Individual values shown. The horizontal line represents median.
