# Supplementary material for: A Checkpoint Reversal Receptor Mediates Bipartite Activation and Enhances CAR T-cell Function
Source: Cancer Res Commun. 2025 Mar 31;5(3):527–48. doi: 10.1158/2767-9764.CRC-24-0125 (PMC11955954; doi:10.1158/2767-9764.CRC-24-0125)
Supplement: Supplementary Figure 11 — Immunohistochemistry and histopathology findings in a metastatic osteosarcoma model after treatment with CARζ/CPR41BB cells. [file crc-24-0125_supplementary_figure_11_suppsf11.pdf]

(A)

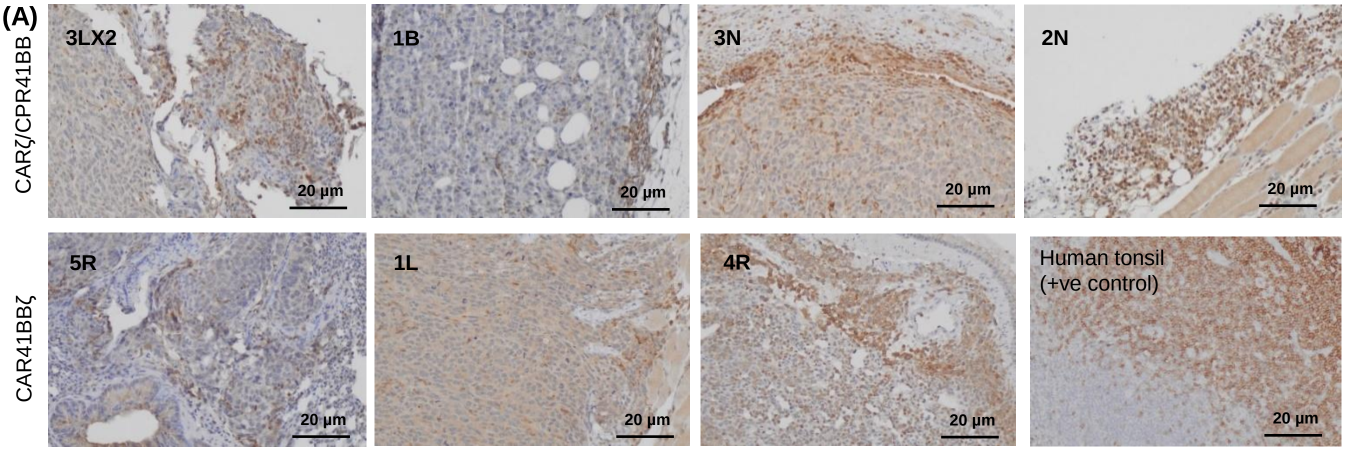

(C)

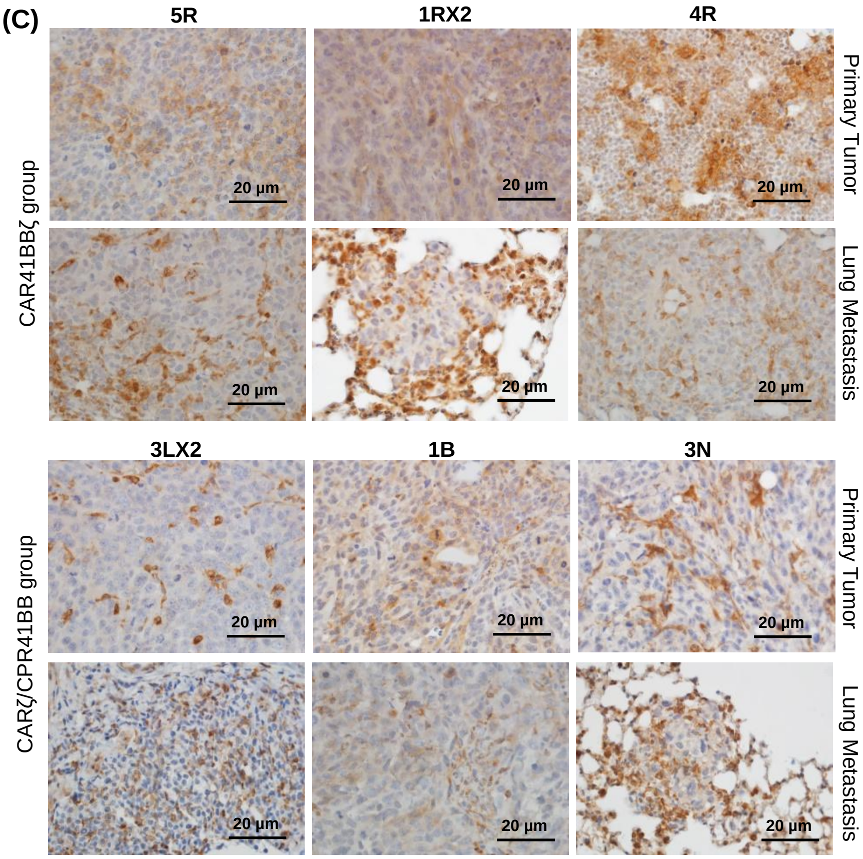

(B)

Treatment Group	Human CD3+ (individual cells)	IHC Score
CAR41BBζ		
5R	Rare to infrequent	1-2
1L	Rare to infrequent	1-2
4R	Infrequent	2
CARζ/CPR41BB		
3LX2	Occasional	3
1B	Rare	1
3N	Occasional to frequent	3-4
2N	Rare to infrequent	1-2

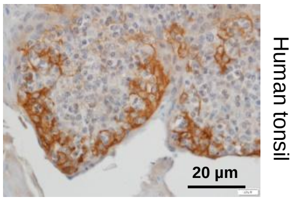

(D)

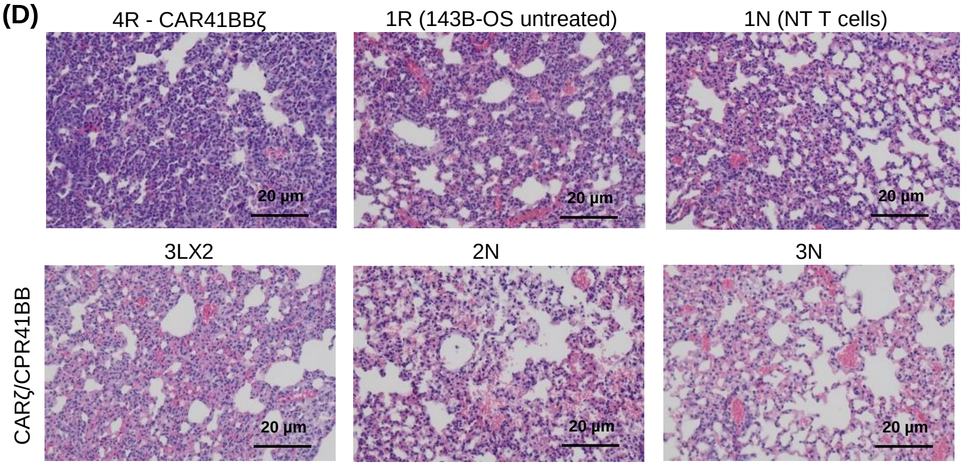

Supplementary Figure 11

Supplementary Figure 11: Immunohistochemistry and histopathology findings in a metastatic osteosarcoma model after treatment with CARζ/CPR41BB cells. (A) and **(B)** Frequency of human $CD3^{+}$ T cells in primary tumors $\geq$2 weeks after intraperitoneal injection of CART as determined by immunohistochemistry. Human tonsil tissue was used as a control. **(C)** Immunohistochemistry confirming PD-L1 expression in primary tumors and lung metastasis collected from mice with 143B osteosarcoma xenografts after treatment with CAR41BBζ (*upper panel*) or CARζ/CPR41BB (*lower panel*) cells. Human tonsil tissue (*lower right panel*) used as control. Representative results from 3 mice in each group shown. **(D)** Hematoxylin and eosin (H&E) staining of lung tissue collected from mice with 143B osteosarcoma xenografts after treatment with CARζ/CPR41BB (*bottom three panels*), CAR41BBζ (*upper left panel*), non-transduced (NT; *upper right panel*), and from untreated (*upper middle panel*) mice. In CARζ/CPR41BB-treated mice, most of the alveoli and airspaces were normal with focal mild interstitial space expansion by lymphocytes. No capillary congestion or interstitial fibrosis. Findings were similar in CAR41BBζ-treated mice. Marked interstitial space expansion, focal capillary congestion, and red blood cell (RBC) extravasation was noted in lung tissue from un-treated mice. All images shown are at 200x magnification.
