# Supplementary material for: A Checkpoint Reversal Receptor Mediates Bipartite Activation and Enhances CAR T-cell Function
Source: Cancer Res Commun. 2025 Mar 31;5(3):527–48. doi: 10.1158/2767-9764.CRC-24-0125 (PMC11955954; doi:10.1158/2767-9764.CRC-24-0125)
Supplement: Supplementary Table 1 — Summary of CAR and CPR constructs evaluated in functional assays [file crc-24-0125_supplementary_table_1_suppst1.pdf]

Supplementary Table 1: Summary of CAR and CPR constructs evaluated in functional assays

Construct name	Full-length name	Description
**CARζ**	FRP5.ζ	1st generation HER2 CAR
**CAR28ζ**	FRP5.CD28.ζ	2nd generation HER2 CAR with CD28 co-stimulation
**CAR41BBζ**	FRP5.41BB.ζ	2nd generation HER2 CAR with 41BB co-stimulation
**CPR28**	PD1.CD28	Checkpoint reversal receptor: native PD-1 ectodomain with CD28 endodomain
**CPR41BB**	PD1.41BB	Checkpoint reversal receptor: native PD-1 ectodomain with 41BB endodomain
**CAR28ζ/PD1$_{TR}$**	FRP5.CD28.ζ.2A.PD1	2nd generation HER2 CAR with native PD-1 ectodomain and truncated PD-1 endodomain (at AA222)
**CARζ/CPR28**	FRP5.ζ.2A.PD1.CD28	1st generation HER2 CAR co-expressed with CPR28
**CARζ/CPR41BB**	FRP5.ζ.2A.PD1.41BB	1st generation HER2 CAR co-expressed with CPR41BB
**CAR28ζ/CPR28**	FRP5.CD28.ζ.2A.PD1.CD28	2nd generation HER2 CAR with CD28 co-stimulation co-expressed with CPR28
**CAR28ζ/CPR41BB**	FRP5.CD28.ζ.2A.PD1.41BB	2nd generation HER2 CAR with CD28 co-stimulation co-expressed with CPR41BB
